# Lemon‐Derived Extracellular Vesicles Nanodrugs Enable to Efficiently Overcome Cancer Multidrug Resistance by Endocytosis‐Triggered Energy Dissipation and Energy Production Reduction

**DOI:** 10.1002/advs.202105274

**Published:** 2022-02-20

**Authors:** Qian Xiao, Wei Zhao, Chentian Wu, Xuejiao Wang, Jianping Chen, Xiubo Shi, Suinan Sha, Jinheng Li, Xiaomei Liang, Yulu Yang, Haoyan Guo, Ying Wang, Jun‐Bing Fan

**Affiliations:** ^1^ Cancer Research Institute Experimental Education/Administration Center School of Basic Medical Sciences Southern Medical University Guangzhou 510515 P. R. China; ^2^ Division of Vascular and Interventional Radiology Department of General Surgery, Nanfang Hospital Southern Medical University Guangzhou Guangdong 510515 P. R. China; ^3^ Department of Hepatobiliary Surgery II Zhujiang Hospital Southern Medical University Guangzhou 510515 P. R. China

**Keywords:** biomimetic nanodrugs, endocytosis, energy dissipation, lemon‐derived extracellular vesicles, multidrug resistance

## Abstract

Multidrug resistance remains a great challenge for cancer chemotherapy. Herein, a biomimetic drug delivery system based on lemon‐derived extracellular vesicles (EVs) nanodrugs (marked with heparin‐cRGD‐EVs‐doxorubicin (HRED)) is demonstrated, achieving highly efficient overcoming cancer multidrug resistance. The HRED is fabricated by modifying functional heparin‐cRGD (HR) onto the surface of EVs and then by loading with doxorubicin (DOX). The obtained HRED enable to effectively enter DOX‐resistant cancer cells by caveolin‐mediated endocytosis (main), macropinocytosis (secondary), and clathrin‐mediated endocytosis (last), exhibiting excellent cellular uptake capacity. The diversified endocytosis capacity of HRED can efficiently dissipate intracellular energy and meanwhile trigger downstream production reduction of adenosine triphosphate (ATP), leading to a significant reduction of drug efflux. Consequently, they show excellent anti‐proliferation capacities to DOX‐resistant ovarian cancer, ensuring the efficiently overcoming ovarian cancer multidrug resistance in vivo. The authors believe this strategy provides a new strategy by endocytosis triggered‐energy dissipation and ATP production reduction to design drug delivery system for overcoming cancer multidrug resistance.

## Graphical abstract

A biomimetic drug delivery system based on lemon‐derived extracellular vesicle (EV) nanodrugs is reported, achieving highly efficiently overcoming cancer multidrug resistance. The lemon‐derived EVs nanodrugs exhibit diverse routes to enter doxorubicin‐resistant cancer cells, enabling to effectively dissipate intracellular energy and trigger the production reduction of adenosine triphosphate. Consequently, they greatly reduce the drug efflux, efficiently overcoming cancer multidrug resistance.

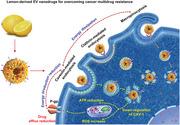

## 1 Introduction

Multidrug resistance remains a major cause for the failure of cancer chemotherapy.^[^ ^1^, ^2^ ^]^ The most popular mechanism of multidrug resistance is associated with the overexpression of resistant protein of P‐glycoprotein (P‐gp), which is an adenosine triphosphate (ATP)‐binding cassette efflux transporter, enabling drugs to pump‐out from cancer cells by utilizing energy from ATP hydrolysis.^[^ ^3^, ^4^, ^5^ ^]^ To date, many efforts, including use of microRNA,^[^ ^6^, ^7^, ^8^ ^]^ RNA interference,^[^ ^9^, ^10^ ^]^ P‐gp inhibitors,^[^ ^11^ ^]^ and nanodrugs,^[^ ^12^ ^]^ have been attempted to overcome cancer multidrug resistance by down‐regulating the expression of P‐gp. However, continually down‐regulating P‐gp may cause dysfunction of normal tissues because P‐gp is also expressed in many normal tissues (such as intestines, kidney, liver, adrenal gland, blood‐brain barrier, and placenta).^[^ ^13^, ^14^ ^]^ In nature, living system is incessantly proceeding cargo delivery for maintaining life activities, through which energy plays a critical role in cargo uptake and output. Alternatively, based on the mechanism of multidrug resistance, ATP as an energy unit is also critically needed in the P‐gp‐mediated drug efflux for multidrug resistance. Inhibiting the production of ATP may be a promising strategy to overcome cancer multidrug resistance.^[^ ^15^, ^16^ ^]^ More recently, ATP‐assistant strategies by targeting mitochondria are also developed to overcome cancer multidrug resistance.^[^ ^17^, ^18^ ^]^ For example, silica‐carbon nanoparticles modified with lipid membrane enable to target mitochondria and produce reactive oxygen species (ROS) under near‐infrared laser, which would efficiently reduce the expression of P‐gp and the amount of ATP to overcome multidrug resistance.^[^ ^18^ ^]^ However, these strategies remained accompanied with dramatic down‐regulation of P‐gp expression. Therefore, ideal strategies with capability of energy dissipation and maintaining the normal expression of P‐gp for overcoming cancer multidrug resistance are urgently expected.

**Scheme 1:**
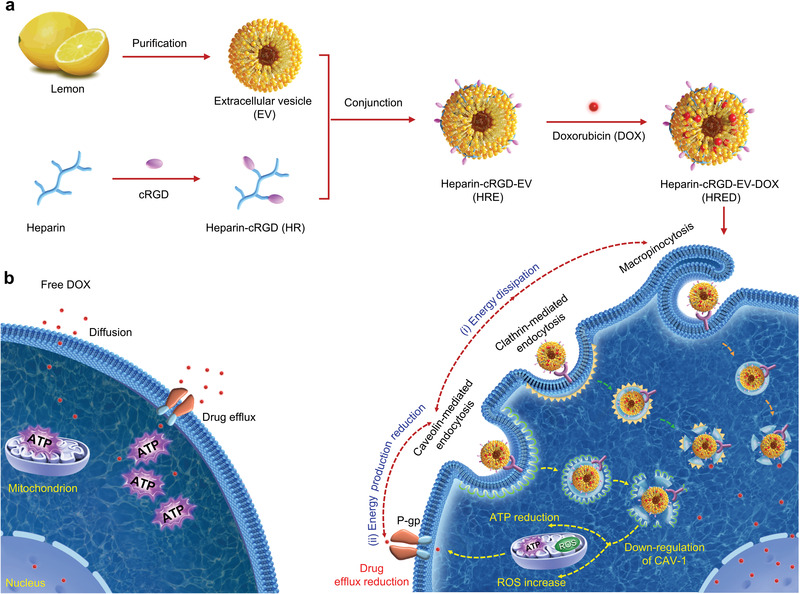
a) schematic illustration of lemon‐derived extracellular vesicle (EV) nanodrugs for overcoming cancer multidrug resistance. The lemon‐derived EV nanodrugs (marked with heparin‐cRGD‐EVs‐doxorubicin (HRED)) were fabricated by modifying heparin‐cRGD (HR) onto the surface of EVs and then by loading with doxorubicin (DOX). The HRED nanodrugs enabled to effectively enter DOX‐resistant cancer cells by caveolin‐mediated endocytosis, macropinocytosis, and clathrin‐mediated endocytosis, exhibiting excellent cellular uptake capacity. They diversified endocytosis capacity enabled to dissipate the intracellular energy. Meanwhile, guided by caveolin‐mediated endocytosis, they could also further down‐regulate the expression of intracellular caveolin‐1 (CAV‐1) to reduce ATP production and increase reactive oxygen species (ROS) level. Thus, combing with the endocytosis‐triggered energy dissipation and ATP production reduction, our HRED nanodrugs would greatly reduce drug efflux, ensuing efficiently overcoming cancer multidrug resistance. b) schematic illustration of free DOX could be effectively pumped out from cancer cells by utilizing the P‐glycoprotein (P‐gp) and the ATP hydrolysis energy.

Herein, we demonstrated a lemon‐derived extracellular vesicles (EVs)‐based drug delivery system that enabled to greatly dissipate intracellular energy triggered by excellent endocytosis, achieving highly efficiently overcoming cancer multidrug resistance. In our designing, we engineered heparin‐cRGD (HR) onto the surface of lemon‐derived EVs to deliver doxorubicin (DOX) for the fabrication of HRE‐DOX (HRED). The introduction of HR enabling HRED has good anti‐complement activation and targeting capacity. The HRED exhibited diverse routes to readily enter DOX‐resistant cancer cells mainly by caveolin‐mediated endocytosis, clathrin‐mediated endocytosis, and macropinocytosis, which enabled to greatly dissipate intracellular energy. Meanwhile, during the downstream delivery guided by caveolin‐mediated endocytosis, our HRED enabled to down‐regulate the expression of caveolin‐1 (CAV‐1) to reduce ATP production and increase ROS level. Thus, combing with the endocytosis triggered‐energy dissipation and caveolin‐mediated endocytosis triggered ATP production reduction and ROS increase, our HRED enabled to efficiently overcome cancer multidrug resistance (**Scheme** **1**).

## 2 Results and Discussion

**Figure 1:**
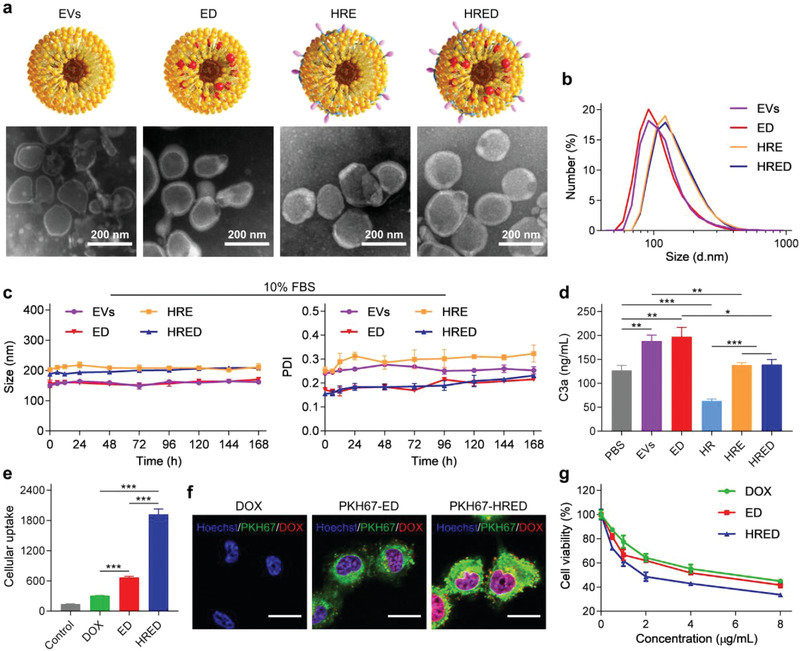
Characterization and in vitro biological activity of lemon‐derived HRED nanodrugs. a) Transmission electron microscopy (TEM) images of lemon‐derived EVs, ED, HRE, and HRED. b) The size distribution of lemon‐derived EVs, ED, HRE, and HRED. c) The stability of EVs, ED, HRE, and HRED in 10% FBS at different time points. d) C3a concentration of human plasma induced by lemon EVs, ED, HR, HRE, and HRED using PBS as negative control. e) The cellular uptake of DOX, ED, and HRED (2.5 µg mL^−1^ DOX) in SKOV3/DOX cells as detected by flow cytometry. Results are represented as mean ± SD (*n* = 3). f) The intracellular localization of DOX, PKH67‐ED, and PKH67‐HRED (2.5 µg mL^−1^ DOX) in SKOV3/DOX cells as detected by confocal microscope. Scale bar: 20 μm. g) Cell viability of SKOV3/DOX cells treated with DOX, ED, and HRED for 48 h. Results are represented as mean ± SD (*n* = 5). ^*^ *p* < 0.05, ^**^ *p* < 0.01, and ^***^ *p* < 0.001.

Recent years, many types of EVs have been developed as drug delivery carriers due to their unique advantages, such as good biocompatibility, great stability, and high intracellular uptake ability. Although cancer cells‐derived EVs possess great homing capability, the procedure was complex and low yield for preparing anti‐cancer drug delivery system.^[^ ^19^, ^20^ ^]^ In comparison, plant‐derived EVs, such as grapefruit, lemon, and ginger, could be served as excellent candidates for delivering various therapeutic agents in view of their large‐scale production and simple encapsulation procedure.^[^ ^21^ ^]^ In particular,because lemon exhibits a certain extend of anti‐tumor effect,^[^ ^22^, ^23^ ^]^ thus we designed the lemon‐derived EVs as carriers to construct biomimetic drug delivery system. In brief, lemon‐derived EVs were isolated from fresh juice and subsequently were modified with HR to prepare HR‐EVs (HRE) by the reaction between amino groups of EVs and carboxyl groups of heparin in HR (Figure S1, Supporting Information). To obtain the optimized ratio of HR and EVs, we detected the size distribution and cellular uptake efficiency of HRE by varying different amount of HR (Figure S2, Table S1, Supporting Information). Among them, HRE2 presented the optimized size distribution and intracellular uptake ability in DOX‐resistant ovarian cancer cells (SKOV3/DOX) and thus HRE2 were used for subsequent experiments (marked as HRE). Subsequently, DOX was encapsulated into HRE to fabricate HRED nanodrugs. To facilitate comparison, we also fabricated EVs‐DOX (ED) nanodrugs by encapsulating DOX into EVs. Transmission electron microscopy (TEM) images showed a typical vesicle structure of EVs, ED, HRE, and HRED with lipid bilayers (**Figure** **1**a). The sizes of EVs, ED, HRE, and HRED were 151.63 ± 5.20, 155.8 ± 12.71, 201.70 ± 5.07, and 202.20 ± 6.21 nm, respectively (Figure 1b; Table S2, Supporting Information). Western blotting result suggested the lemon‐derived EVs were enriched in protein markers of EVs (Alix, TSG101, and CD81), which were also high expression in cells (Figure S3, Supporting Information). UV–vis spectra indicated that ED and HRED had a maximum absorption peak at 490 nm in accordance with DOX, suggesting that DOX was successfully encapsulated into the EVs and HRE (Figure S4, Supporting Information). It was detected that 1 µg (protein amount) of EVs contained 1.35 ± 0.04 µg of DOX and 0.44 ± 0.01 µg of cRGD in HRED determined by UV–vis spectra and BCA assay, respectively (Table S3, Supporting Information). The entrapment efficiency and loading efficiency of DOX is shown in Table S4, Supporting Information. Furthermore, to investigate the stability of obtained HRED, we selected 10% fetal bovine serum (FBS) solution to mimic blood condition and the result showed the sizes and polydispersity index (PDI) of EVs, ED, HRE, and HRED had no significant change within 168 h, indicating that they had good stability (Figure 1c). The complement activation capacity of nanodrugs is an important parameter to evaluate their fate by phagocyte removal.^[^ ^24^, ^25^ ^]^ We investigated the anti‐complement capacity of HRED and the result demonstrated that the obtained HRED possessed good anti‐complement activation by inhibiting C3a formation to escape phagocytosis (Figure 1d). We then investigated the released behavior of ED and HRED in PBS (pH = 7.4) conditions (Figure S5, Supporting Information). It showed that ED and HRED could liberate about 40% in 48 h under PBS (pH = 7.4) condition and presented similar release rate under physiological condition. Next, we selected DOX‐resistant ovarian cancer cells (SKOV3/DOX), DOX‐sensitive ovarian cancer cells (SKOV3), Taxol‐resistant ovarian cancer cells (A2780/Taxol), Taxol‐sensitive ovarian cancer cells (A2780), and Cisplatin‐resistant ovarian cancer cells (SKOV3/Cisplatin) to investigate the biological activities of HRED in vitro. The expression of integrin *α*v*β*3 and resistant protein P‐gp in cells were determined by western blotting. In our cell model, both DOX‐resistant and DOX‐sensitive ovarian cancer cells (SKOV3/DOX and SKOV3) presented high expression of *α*v*β*3, while Taxol‐resistant and Taxol‐sensitive ovarian cancer cells (A2780/Taxol and A2780) only presented little expression of *α*v, and only resistant cells SKOV3/DOX, A2780/Taxol, and Cisplatin‐resistant ovarian cancer cells (SKOV3/Cisplatin) had high P‐gp expression (Figure S6, Supporting Information). We used flow cytometry to detect the cellular uptake of PKH67‐labeled EVs (PKH67‐EVs), PKH67‐labeled HRE (PKH67‐HRE), DOX, ED, and HRED in SKOV3/DOX and SKOV3 cells. The results indicated that compared to free DOX and ED, the HRED showed excellent intracellular uptake capacity. In particular, PKH67‐HRE and HRED by HR modification would obviously enhance the intracellular uptake (Figure 1e,f; Figures S7,S8, Supporting Information). According to the intracellular trafficking of EVs,^[^ ^26^, ^27^, ^28^, ^29^ ^]^ HRED and ED entered into lysosomes or cytoplasm after they internalized into cells, thus some of DOX from HRED or ED was degraded in lysosomes, and other DOX was released into the cytoplasm. Laser confocal microscopy images of ED and HRED in SKOV3/DOX and SKOV3 cells also showed that EVs or HRE (PKH67 labeled, green fluorescence) located in cytoplasm and red signal of DOX mainly distributed in nucleus. Then, we investigated the anti‐proliferation effects of free DOX, ED, and HRED in SKOV3/DOX and SKOV3 cells using MTT assays. All treatment groups (DOX, ED, and HRED) exhibited dose‐dependent inhibitory effect in SKOV3/DOX and SKOV3 cells. Compared to DOX and ED, HRED showed the best anti‐proliferation in SKOV3/DOX and SKOV3 cells (Figure 1g; Figure S9, Table S5, Supporting Information). Furthermore, we evaluated the sensitivity of our HRED to other resistant ovarian cancer cells (SKOV3/Cisplatin and A2780/Taxol) with high P‐gp expression (Figure S9, Table S5, Supporting Information). It showed that HRED also enhanced the cytotoxicity to SKOV3/Cisplatin and A2780/Taxol cells in comparison with free DOX, indicating our HRED presented the validity to different resistance cancer cells. In addition, we prepared EVs or HRE encapsulated with Taxol (EVs‐Taxol (ET) and heparin‐cRGD‐EVs‐Taxol (HRET)), and evaluated their cytotoxicity to A2780/Taxol cells (Figure S9, Table S6, Supporting Information). It showed HRET and ET exhibited higher cytotoxicity to A2780/Taxol cells in compared with free Taxol and the cytotoxicity of HRET was higher than that of ET, suggesting HRE would be good vehicles in the treatment of multidrug resistant tumors. Flow cytometry analysis further verified the apoptotic effect induced by the composition of HRED for SKOV3/DOX (Figure S10, Supporting Information). Compared with lemon‐derived EVs, the apoptotic effect of HRE was significantly enhanced after the introduction of tumor‐targeting effect, and HRED presented the strongest apoptotic effect for SKOV3/DOX cells. Also, we assayed the effect of our nanodrug on cell cycle by flow cytometry (Figure S11, Supporting Information). It showed that EVs and HRE could block the cell cycle in the S phase, while ED and HRED could induce cell cycle arrest at the G2/M phase after the introduction of DOX. The results were in accordance with the effect of lemon‐derived EVs and DOX on the cell cycle.^[^ ^23^, ^30^ ^]^ Because apoptosis is often accompanied by a decrease in mitochondrial membrane permeabilization, we next detected their mitochondrial membrane potential (Figure S12, Supporting Information). The results showed that both EVs and HRE triggered the deceased mitochondrial membrane potential while they had no obvious difference. Interestingly, compared with EVs and HRE, ED, and HRED could not induce significantly decreased mitochondrial membrane potential. It was explained the mitochondrial membrane potential was possibly disturbed by the auto‐fluorescence of DOX. These results demonstrated that HRED nanodrugs with capacity of excellent intracellular uptake and anti‐proliferation could be successfully fabricated.

**Figure 2:**
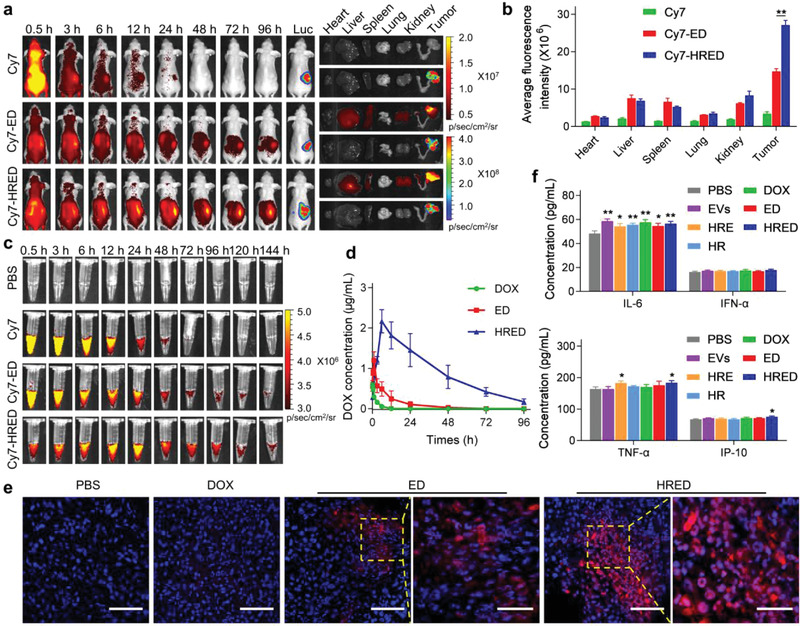
Biodistribution, tumor targeting ability, pharmacokinetic properties, and immunogenicity of HRED nanodrugs. a) In vivo fluorescence images of orthotopic SKOV3/DOX‐Luc ovarian cancer xenograft nude mouse model at different time points after intraperitoneal injection of free Cy7, Cy7‐ED, and Cy7‐HRED. After 96 h, mice were sacrificed and their major organs and tumors were collected to detect Cy7 and Luc signals. b) The corresponding semiquantitative results of ex vivo organs and tumors in different groups. c) Fluorescent images of rat plasma with free Cy7, Cy7‐ED, and Cy7‐HRED injected to rats at different time points, as determined by in vivo imaging system (IVIS). d) DOX concentrations in rat plasma were detected at different time points after DOX, ED, and HRED were injected in rat via intraperitoneal administration. e) Confocal images of the ovarian cancer tissues from orthotopic SKOV3/DOX‐Luc ovarian cancer xenograft nude mouse model after intraperitoneal injection of PBS, DOX, ED, and HRED for 24 h. Nucleus stained by Hoechst 33342 and red represented DOX. Scale bar: 50 µm in low magnification, 20 µm in high magnification. f) Cytokine (IL6, IFN‐*α*, TNF‐*α*, and IP‐10) concentrations of rat serum at 24 h after administration of PBS, EVs, HRE, HR, DOX, ED, and HRED via intraperitoneal injection, as measured by ELISA. Results are represented as mean ± SD (*n* = 3). ^*^ *p* < 0.05, ^**^ *p* < 0.01, and ^***^ *p* < 0.001.

The in vivo biodistribution and cancer targeting capacity of Cy7 labeled‐ED (Cy7‐ED) and Cy7 labeled‐HRED (Cy7‐HRED) were investigated in the orthotopic Luciferase‐expressing SKOV3/DOX (SKOV3/DOX‐Luc) cells ovarian cancer xenograft nude mouse model via intraperitoneal injection. Real‐time images of biodistributions of free Cy7, Cy7‐ED, and Cy7‐HRED were observed at different time after administration, through which tumor was monitored by bioluminescence of SKOV3/DOX‐Luc cells. The results showed the fluorescent intensity of Cy7‐HRED was obviously higher than that of free Cy7 and Cy7‐ED at 96 h after administration (**Figure** **2**a). To further evaluate their biodistributions, mice were sacrificed at 96 h after injection and the main organs (heart, liver, spleen, lung, kidney, and tumor) were carefully excised to compare the levels of fluorescence accumulation. The results showed that Cy7‐HRED nanodrugs were mainly distributed in liver, spleen, kidney, and tumor and its fluorescence intensity at tumor site was much higher than Cy7‐ED (Figure 2a,b), suggesting that our HRED nanodrugs had good cancer targeting. Similar results observed in orthotopic Luciferase‐expressing SKOV3 (SKOV3 ‐Luc) cells ovarian cancer xenograft nude mouse model (Figure S13, Supporting Information). At the same time, we tested the retention of Cy7, Cy7‐ED, and Cy7‐HRED in the blood of Sprague‐Dawley (SD) rats. The results indicated that Cy7, Cy7‐ED, and Cy7‐HRED were observed in plasma, and the fluorescent signal of Cy7‐HRED nanodrugs remained detected until 144 h, which was much higher than Cy7 and Cy7‐ED groups (Figure 2c; Figure S14, Supporting Information). It was possibly attributed to the anti‐complement activation ability of heparin. We then detected DOX concentration in rat plasma at different time points after intraperitoneal injection of DOX, ED, and HRED (Figure 2d; Table S7, Supporting Information). The maximal concentration and half‐life of DOX in HRED was significantly higher than that in DOX and ED, and DOX in HRED went through longer elimination time with half‐life about 24 h. Next, we assessed the accumulation of DOX, ED, and HRED in tumor tissues of orthotopic SKOV3/DOX‐Luc and SKOV3‐Luc ovarian cancer xenograft nude mouse model by laser confocal microscopy. It was detected that DOX hardly accumulated in DOX‐resistant ovarian cancer, while HRED exhibited excellent accumulation capacity (Figure 2e). The accumulation of HRED nanodrugs was much higher than that of ED in DOX‐resistant ovarian cancer and DOX‐sensitive ovarian cancer (Figure 2e; Figure S15, Supporting Information). To evaluate the biosafety and biocompability of HRED, we investigated their hemolysis, inflammatory cytokine, and biochemical indicators in mouse serum after treatment. The analysis result shows EVs and HRE almost did not induce hemolysis compared with water and PBS (Figure S16, Supporting Information). Although HRED nanodrugs induced a slight increase of inflammatory cytokine (IL‐6, IFN‐*α*, TNF‐*α*, and IP‐10), it was tolerant to their increased level in vivo (Figure 2f). The biochemical indicators (alanine aminotransferase (ALT), aspartate aminotransferase (AST), blood urea nitrogen (BUN) and creatinine (Cre)) related to liver and kidney functions in EVs, ED, and HRED presented similar level as control (Figure S17, Supporting Information). The results indicated our HRED had good biosafety and biocompability in vivo. Collectively, our HRED exhibited excellent long‐term retention time, cancer targeting capacity, and biosafety in vivo, affording effective accumulation in DOX‐resistant ovarian cancer.

**Figure 3:**
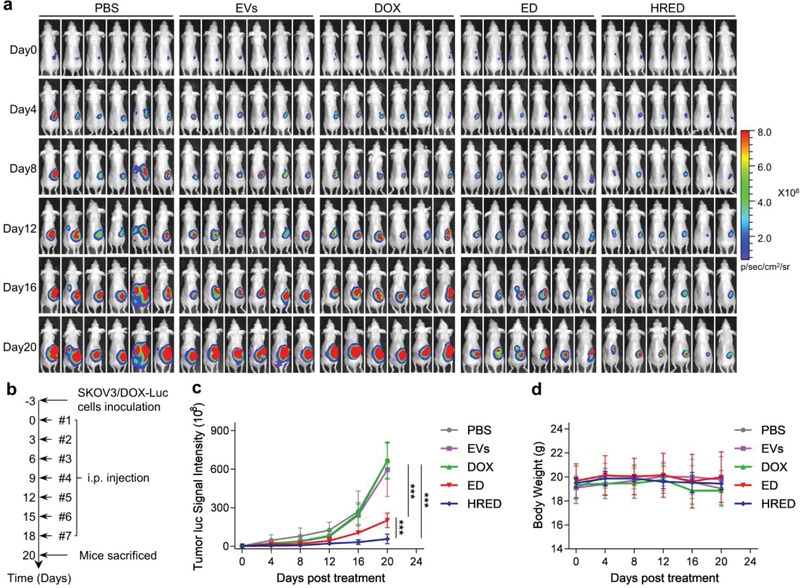
Anti‐tumor effect of EVs, DOX, ED, and HRED in the orthotopic SKOV3/DOX‐Luc ovarian cancer xenograft nude mouse model. a) IVIS bioluminescent imaging of orthotopic SKOV3/DOX‐Luc ovarian cancer xenograft nude mouse from each group during treatment. All mice were injected SKOV3/DOX‐Luc cells into the ovaries, followed by treatment and measurement of bioluminescence after 3 days (day 0). The mice of the control group were intraperitoneally treated with PBS and those in the other groups with EVs (the equivalent dose with HRED), free DOX, ED, or HRED (2.5 mg kg^−1^ DOX) every 3 days. The tumor size was quantified by IVIS every 4 days. b) Protocol for tumor implantation and treatment used in this study. c) The luminescent signal intensity of the mice in all groups. d) Body weight of the mice in all groups. Results are represented as mean ± SD (*n* = 6). ^*^ *p* < 0.05, ^**^ *p* < 0.01, and ^***^ *p* < 0.001.

**Figure 4:**
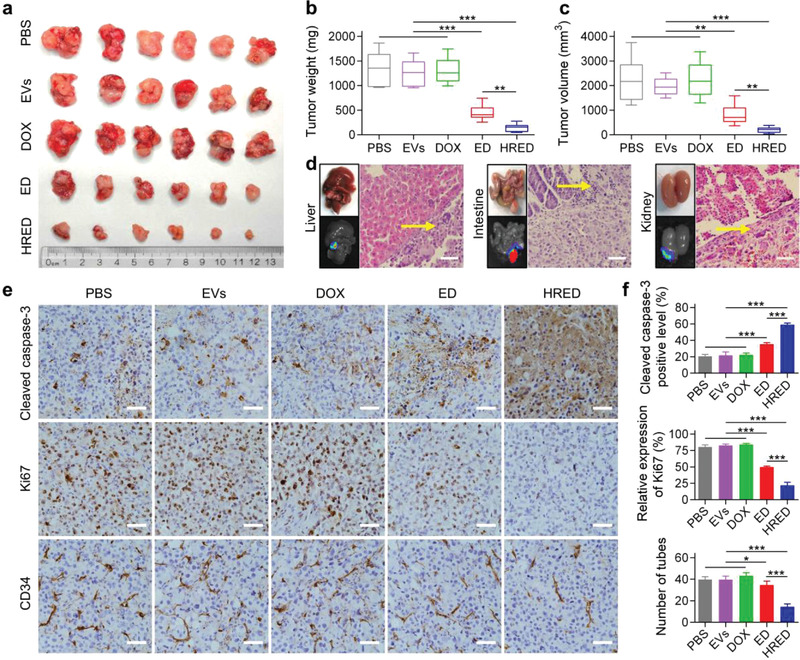
Anti‐tumor effect of EVs, DOX, ED, and HRED in the SKOV3/DOX‐Luc orthotopic ovarian cancer xenograft nude mice model. a) Excised tumor of the mice from each group. Scale: 1 unit = 1 cm. b) Tumor weights of the mice in all groups. c) Tumor volume of the mice in all groups. d) Excised organ images and H&E staining of metastatic nodules of liver in PBS group, intestine in EVs group, and kidney in DOX group. yellow arrows point to tumor metastasis. Scale bar: 50 µm. e) Ovarian cancer tumors from different treatment groups were immunostained with cleaved caspase‐3 for cell apoptosis, with Ki67 for cell proliferation, and with CD34 for the detection of endothelial‐lined vessels. Scale bars, 50 µm. f) The immunohistochemical corresponding quantitative results of cleaved caspase‐3, Ki67, and CD34 in different groups. Results are represented as mean ± SD (*n* = 6). ^*^ *p* < 0.05, ^**^ *p* < 0.01, and ^***^ *p* < 0.001.

The antitumor efficacy and systemic toxicity of HRED on orthotopic SKOV3‐Luc ovarian cancer xenograft nude mouse model and orthotopic SKOV3/DOX‐Luc ovarian cancer xenograft nude mouse model in vivo were subsequently evaluated, respectively. In orthotopic SKOV3‐Luc ovarian cancer xenograft nude mouse model, both PBS and lemon‐derived EVs could not inhibit tumor growth though lemon‐derived EVs presented a certain extend of anti‐proliferation effect (Figure S18, Supporting Information); compared to ED and DOX, a significant suppression on tumor growth and metastasis was observed in mice treated with HRED. The immunohistochemical results showed that HRED exhibited a more significant antitumor effect than DOX and ED. In addition, hematoxylin and eosin (H&E) staining of the main organs showed that there was no obvious tissue damage except for the metastases nodules in the liver, spleen, and kidney of PBS, and liver of DOX group (Figures S19–S22, Supporting Information). On the basis of these results, we next investigated the antitumor efficacy of EVs, DOX, ED, and HRED in orthotopic SKOV3/DOX‐Luc ovarian cancer xenograft nude mouse model. The tumor growth was measured by monitoring luminescence intensity via in vivo imaging system (IVIS). The results demonstrated that free DOX presented almost no inhibitory effect on tumor growth, indicating orthotopic SKOV3/DOX‐Luc ovarian cancer xenograft nude mouse model was resistant to DOX treatment. In contrast, HRED exhibited significant tumor inhibition effect in comparison with PBS, EVs, free DOX, and ED (*p* < 0.001) (**Figure** **3**a–c). Simultaneously, we monitored body weight in all groups to estimate their potential adverse effects and the result showed the body weight of mice remained stable during the process of treatment period (Figure 3d). After 20 days of treatment, mice were sacrificed and the tumors were excised for further evaluation. The average primary tumor weight and volume treated by HRED (weight, 143.57 ± 83.88 mg; volume, 203.09 ± 121.06 mm^3^) were significantly smaller than those of PBS (weight, 1345.65 ± 352.00 mg; volume, 2225.82 ± 896.38 mm^3^), EVs (weight, 1261.87 ± 269.01 mg, volume, 1964.43 ± 373.92 mm^3^), DOX (weight, 1301.72 ± 256.04 mg; volume, 2240.69 ± 760.33 mm^3^), and ED (weight, 448.53 ± 163.87 mg, volume, 816.92 ± 429.67 mm^3^) (**Figure** **4**a–c). Moreover, it was observed a large amount of bloody ascites in the peritoneal cavities of mice treated with PBS, EVs, and DOX, while the ED group exhibited less bloody ascites. Remarkably, mice treated with HRED nearly had no obvious ascites in the peritoneal cavities (Figure S23, Supporting Information). We subsequently examined the metastases in the main organs (heart, liver, spleen, lung, kidney, and intestine) via observation and bioluminescence detection. The results showed visible metastatic nodules could be detected in the livers of PBS group, intestines of EVs group, and kidneys of DOX group, but were not observed in ED group and HRED group (Figure S24, Supporting Information). H&E staining results confirmed the presence of tumor cells in these metastatic nodules (Figure 4d). In order to verify the suppression mechanisms, we performed immunohistochemical analysis for cleaved caspase‐3 (marker for cellular apoptosis), Ki67 (marker for cellular proliferation), and CD34 (marker for the endothelial‐lined vessels) (Figure 4e,f). It was shown that the cleaved caspase‐3 level of HRED was higher than that of PBS, EVs, DOX, and ED, and the order of the cleaved caspase‐3 positive rate was as follows: HRED (59.10 ± 1.94%) > ED (35.40 ± 1.90%) > DOX (22.13 ± 2.52%), EVs (21.74 ± 4.10%), and PBS (20.49 ± 2.43%). The least number of Ki67‐positive cells was observed in the tumors treated with HRED (21.82 ± 4.74%), followed by the ED group (49.66 ± 1.65%), while there was no obvious difference between PBS, EVs, and DOX groups (80.45 ± 3.17%, 82.67 ± 2.25%, and 83.87 ± 1.94%, respectively). The results suggested HRED effectively promoted apoptosis and inhibited cell proliferation in DOX‐resistant ovarian cancer tumors. In addition, the number of tubes consisting of CD34^+^ endothelial‐lined vessels in the HRED (14.4 ± 2.70) groups was significantly lower than that of ED (34.6 ± 3.65), DOX (43.2 ± 3.03), EVs (39.6 ± 3.36), and PBS (39.8 ± 2.59), indicating our HRED also enabled to efficiently inhibit angiogenesis in DOX‐resistant ovarian cancer. Furthermore, H&E staining of the main organs showed that there was no obvious tissue damage except for the metastases nodules in the liver of PBS and EVs groups, and kidney of DOX group (Figure S25, Supporting Information). These results indicated that HRED exhibited significant capacity to suppress tumor growth through apoptosis promotion, anti‐proliferation, as well as anti‐angiogenesis in DOX‐resistant ovarian cancer therapy.

**Figure 5:**
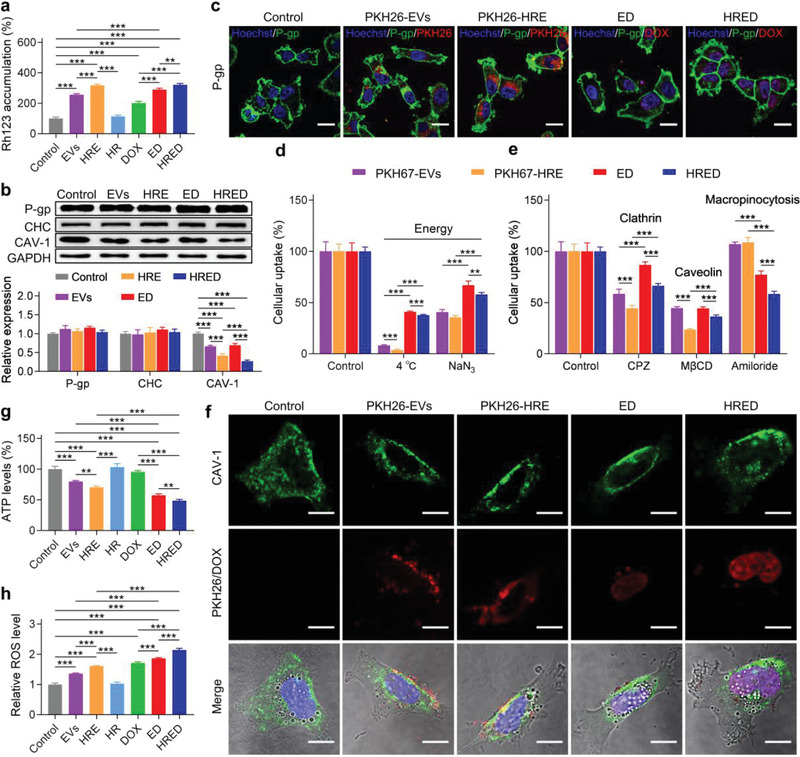
Mechanism of HRED in overcoming ovarian cancer multidrug resistance. a) Relative intracellular accumulation of Rh123 in SKOV3/DOX cells after pre‐treated with EVs, HRE, HR, DOX, ED, and HRED for 24 h, as detected by flow cytometry. b) Protein levels and relative expression of P‐gp, clathrin heavy chain (CHC), and CAV‐1 in SKOV3/DOX cells after being treated with EVs, HRE, ED, and HRED for 24 h. c) The intracellular localization of P‐gp after treated with PKH26‐EVs, PKH26‐HRE, ED, and HRED in SKOV3/DOX cells for 2 h, as determined by using confocal microscopy. Scale bar: 20 μm. d) Effects of temperature and mitochondrial inhibitor sodium azide (NaN_3_) on PKH67‐EVs, PKH67‐HRE, ED, and HRED intracellular uptake. e) Effects of clathrin‐mediated endocytosis inhibitor chlorpromazine (CPZ), caveolin‐mediated endocytosis inhibitor methyl‐*β*‐cyclodextrin (M*β*CD), and macropinocytosis inhibitor amiloride on PKH67‐EVs, PKH67‐HRE, ED, and HRED intracellular uptake. f) The intracellular localization of CAV‐1 after treated with PKH26‐EVs, PKH26‐HRE, ED, and HRED in SKOV3/DOX cells for 2 h, as detected by confocal microscope. Scale bar: 10 μm. g) Relative ATP levels in SKOV3/DOX cells after being treated with EVs, HRE, HR, DOX, ED, and HRED for 24 h. h) Relative reactive oxygen species (ROS) levels in SKOV3/DOX cells after being treated with EVs, HRE, HR, DOX, ED, and HRED for 24 h. Results are represented as mean ± SD (*n* = 3). ^*^ *p* < 0.05, ^**^ *p* < 0.01, and ^***^ *p* < 0.001.

We comprehensively investigated the mechanism of our HRED on overcoming cancer multidrug resistance, including the efflux function and expression of P‐gp and endocytic pathways. We first evaluated the influence of HRED on the efflux function of P‐gp by detecting the intracellular accumulation of P‐gp substrate of rhodamine 123 (Rh123) in SKOV3/DOX cells. The results showed that the concentrations of Rh123 were significantly improved in EVs‐based nanodrugs (EVs, HRE, ED, and HRED) in comparison with HR and DOX, suggesting that they could effectively inhibit the efflux function of P‐gp (**Figure** **5**a). Further, we examined the expression of P‐gp in SKOV3/DOX cells after treating with EVs‐based nanodrugs (EVs, HRE, ED, and HRED), HR, and DOX for 24 h. It showed the expression of P‐gp could not be down‐regulated in SKOV3/DOX cells after treating with EVs‐based nanodrugs (EVs, HRE, ED, and HRED) (Figure 5b; Figure S26, Supporting Information). We used immunofluorescence staining to detect the distribution of P‐gp during the internalization of PKH26‐labeled EVs (PKH26‐EVs), PKH26‐labeled HRE (PKH26‐HRE), ED, and HRED in SKOV3/DOX cells. The result showed that most of P‐gp located in cell membrane and they nearly had no change after internalization of EVs‐based nanodrugs (EVs, HRE, ED, and HRED) (Figure 5c; Figure S27, Supporting Information). These results demonstrated that our EVs‐based nanodrugs (EVs, HRE, ED, and HRED), enabled to effectively inhibit the efflux function of P‐gp, but could not down‐regulate the expression of P‐gp. In other words, our EVs‐based nanodrugs for overcoming cancer multidrug resistance were not dependent on the down‐regulation of P‐gp expression.

It is well known that mitochondrion is energy factory for producing ATP via oxidative respiratory chain, in which a variety of enzymes are involved. Sodium azide (NaN_3_) is an inhibitor of cytochrome c oxidase related to the oxidative respiratory chain, and low temperature enables to lead to a decrease in enzyme activity, both of which can reduce the production of ATP.^[^ ^31^, ^32^, ^33^, ^34^ ^]^ Thus, we first examined the role of energy in the cellular uptake of EVs‐based nanodrugs (EVs, HRE, ED, and HRED) in SKOV3/DOX cells by treating with 4 ℃ or mitochondrial inhibitor NaN_3_. As expected, the results indicated that when SKOV3/DOX cells were incubated at 4 ℃ or treated with mitochondrial inhibitor NaN_3_, the internalization capacity of PKH67‐EVs, PKH67‐HRE, ED, and HRED were dramatically reduced, suggesting that the intracellular uptakes of EVs‐based nanodrugs (EVs, HRE, ED, and HRED) were energy dissipation process (Figure 5d). Subsequently, the endocytic pathways of PKH67‐labled EVs (PKH67‐EVs) and PKH67‐labled HRE (PKH67‐HRE), ED, and HRED in SKOV3/DOX cells were carried out in the presence of endocytosis inhibitors. The results showed that cellular uptake of EVs and HRE mainly proceeded with clathrin‐mediated endocytosis and caveolin‐mediated endocytosis, while cellular uptake of ED and HRED proceeded with clathrin‐mediated endocytosis, caveolin‐mediated endocytosis, and macropinocytosis in SKOV3/DOX cells. Compared to the control, cellular uptake of EVs‐based nanodrugs (PKH67‐EVs, PKH67‐HRE, ED, and HRED) in SKOV3/DOX cells was significantly inhibited in the presence of chlorpromazine (CPZ, clathrin‐mediated endocytosis inhibitor) and methyl‐*β*‐cyclodextrin (M*β*CD, caveolin‐mediated endocytosis inhibitor). In particular, M*β*CD exhibited a stronger inhibitory effect on the cellular uptake of HRED compared with CPZ, revealing cellular uptake of our EVs‐based nanodrugs (EVs, HRE, ED, and HRED) by caveolin‐mediated endocytosis was more efficient than that of clathrin‐mediated endocytosis, especially for targeting modified HRE and HRED. Moreover, the cellular uptake of ED and HRED was distinctly decreased in the presence of amiloride (macropinocytosis inhibitor), suggesting that cellular uptake of ED and HRED also proceeded with macropinocytosis. As shown in Figure 5e, the caveolin‐mediated endocytosis of HRED was more efficient than that of clathrin‐mediated endocytosis and macropinocytosis. In general, most of nanodrugs entering cells are based on clathrin‐mediated endocytosis,^[^ ^35^ ^]^ our HRED combined with caveolin‐mediated endocytosis, clathrin‐mediated endocytosis, and macropinocytosis, exhibiting excellent capacity of intracellular uptake. Meanwhile, the diverse routes of HRED for intracellular uptake enabled to effectively dissipate intracellular energy, which is critical to overcome cancer multidrug resistance.

We measured the typical protein expression participated in the clathrin‐ and caveolin‐mediated endocytosis in SKOV3/DOX cells by treating with EVs‐based nanodrugs (EVs, HRE, ED, and HRED), HR, and DOX for 24 h. Importantly, we found the expression of clathrin heavy chain (CHC) nearly had no change, while CAV‐1 had an obvious down‐regulation after incubated with EVs‐based nanodrugs (EVs, HRE, ED, and HRED) (Figure 5b). While, both HR and DOX could not down‐regulate the expression of CHC and CAV‐1 (Figure S26, Supporting Information). CAV‐1 is the most important structural protein of caveolae in the surface of many mammalian cells,^[^ ^36^, ^37^, ^38^, ^39^ ^]^ which plays critical role in caveolin‐mediated endocytosis (such as cell signaling,^[^ ^40^, ^41^, ^42^ ^]^ lipid regulation^[^ ^43^, ^44^, ^45^, ^46^ ^]^) and subsequent cytoplasmic transportation (vesicular transport^[^ ^47^, ^48^, ^49^, ^50^, ^51^ ^]^). It was reported the down‐regulation of CAV‐1 could promote cholesterol accumulation in mitochondria resulting in mitochondria dysfunction, such as weakening membrane fluidity, reducing ATP, and increasing ROS level.^[^ ^52^, ^53^, ^54^, ^55^, ^56^ ^]^ To verify whether the down‐regulation of CAV‐1 was the result of mRNA transcription change, we detected the mRNA level of CHC and CAV‐1 by q‐PCR assay. The result demonstrated that our EVs‐based nanodrugs (EVs, HRE, ED, and HRED) could not change the mRNA level (Figure S28, Supporting Information), indicating the down‐regulation of CAV‐1 protein level was not attributed to mRNA transcription change but was possibly ascribed to the consumption of CAV‐1 during endocytosis. Subsequently, we observed the co‐localization of CAV‐1 with PKH26 labeled EVs and HRE (PKH26‐EVs and PKH26‐HRE), ED, and HRED in SKOV3/DOX cells. Immunofluorescence staining result indicated that green fluorescence of CAV‐1 was uniformly distributed into cytoplasm in SKOV3/DOX cells. Accompanying with the uptake of EVs‐based nanodrugs, most of CAV‐1 were moved into cellular interior near the nucleus and was co‐localized with PKH26‐EVs and PKH26‐HRE, through which DOX within HRED was delivered into nucleus (Figure 5f; Figure S29, Supporting Information).

On the basis of these results, we next detected the influence of EVs‐based nanodrugs (EVs, HRE, ED, and HRED) on the intracellular ATP concentration in SKOV3/DOX cells. The result showed that EVs‐based nanodrugs (EVs, HRE, ED, and HRED) could effectively reduce the intracellular ATP level, while HR and DOX nearly could not decrease ATP concentration. Compared to the EVs, HRE, and ED, our HRED enabled to significantly decrease ATP concentration (Figure 5g). Furthermore, we investigated ROS level induced by EVs‐based nanodrugs (EVs, HRE, ED, and HRED) because DOX could promote cancer cell apoptosis by increasing ROS level. The result showed that the ROS levels induced by EVs‐based nanodrugs (EVs, HRE, ED, and HRED) were significantly increased in SKOV3/DOX cells and the order of ROS levels was followed: HRED > ED > HRE > EVs (Figure 5h). These results demonstrated that our HRED nanodrugs enabled to effectively dissipate intracellular energy triggered by endocytosis. During the transport process triggered by caveolin‐mediated endocytosis, our HRED nanodrugs could result in dysfunction of mitochondria to reduce ATP production and increase ROS. Thus, our HRED nanodrugs enabled to efficiently overcome cancer multidrug resistance by endocytosis‐triggered energy dissipation and ATP production reduction.

## 3 Conclusion

In summary, we have demonstrated that lemon‐derived HRED enable highly efficient overcoming DOX‐resistant ovarian cancer multidrug resistance. The obtained HRED nanodrugs have excellent cellular uptake capacities and can enter DOX‐resistant cancer cells via clathrin‐mediated endocytosis, caveolin‐mediated endocytosis, and macropinocytosis, which efficiently dissipate the intracellular energy. Meanwhile, guided by caveolin‐mediated endocytosis, our HRED can greatly reduce ATP production and increase ROS. During this process, the efflux function of P‐gp can be effectively inhibited, but the expression of P‐gp is nearly not down‐regulated. Thereby, the obtained HRED nanodrugs exhibit excellent anti‐proliferation ability in vivo to overcome cancer multidrug resistance and metastases of DOX‐resistant ovarian cancer. Thus, combing with the endocytosis‐triggered energy dissipation and caveolin‐mediated endocytosis triggered ATP production reduction, our HRED nanodrugs enable to greatly reduce drug efflux, thereby ensuring efficiently overcome cancer multidrug resistance.

## 4 Experimental Section

### Isolation and Purification of EVs

EVs were isolated from lemon juice as previously described.^[^ ^57^ ^]^ Briefly, lemon juice was collected and differentially centrifuged (500 × *g* for 10 min, 2000 × *g* for 20 min, 5000 × *g* for 30 min, 10 000 × *g* for 1 h, and 100 000 × *g* for 2 h) and the sediments were then purified on a sucrose gradient (8%, 30%, 45%, and 60% sucrose in 20 mm Tris.Cl, pH = 7.2). The concentration of EVs was determined by detecting protein concentration using BCA protein quantification assay kit. Isolated lemon‐derived EVs were characterized with the minimal experimental requirements for EVs.^[^ ^58^ ^]^ The size distribution and Zeta potential of the EVs were measured by dynamic light scattering using a Zetasizer Nano‐Zs (Malvern Instruments, UK). The surface morphology of the EVs was determined by TEM (Hitachi HC‐1, 80 kV) after negative staining with phosphotungstic acid.

### Synthesis of HR

In a typical synthesis, succinylated‐heparin (50 mg), cRGD peptide (6 mg), 1‐ethyl‐3‐(3‐dimethylaminopropyl)carbodiimide hydrochloride (EDC, 8 mg), and N‐hydroxysuccinimide (NHS, 8 mg) were first added into dimethyl sulfoxide (1.5 mL) and subsequently reacted at room temperature for 12 h for the synthesis of HR. Next, the resultant HR product was dialyzed in deionized water using a dialysis membrane (MWCO 3500 Da) for 48 h and then lyophilized. The ^1^H NMR spectra of HR was detected on a NMR spectroscopy (Bruker Biospin, AVANCE III HD 600MHz) to confirm the conjugation of heparin and cRGD peptide.

### Preparation and Characterization of HRED

A convenient and rapid method to endow lemon EVs with tumor‐targeting function was designed. Briefly, 100 µg of EVs and 4 mg of HR were added into 2 mL deionized water and gently agitated at room temperature for 12 h by using EDC (5 mg) and NHS (5 mg) as catalysts to prepare HRE. Then, the obtained product was washed and ultracentrifuged (120 000 × *g*, 1 h). For DOX loading, 250 µg of DOX hydrochloride solution (5 mg mL^−1^) was added to the EVs or HRE solution (1 mL, 100 µg mL^−1^) with moderate stirring at room temperature for 12 h. The suspensions were washed by PBS and then ultracentrifuged twice at 120 000 × *g* for 1 h each time to obtain ED or HRED. The size distribution, Zeta potential, and surface morphology of ED, HRE, and HRED were performed using the same methods as described above. The successful loading of DOX in ED and HRED was detected by UV–vis absorption spectrum via UV–vis spectrophotometer (SHIMADZU, UV‐2600, Tokyo, Japan). The amount of DOX loaded into ED and HRED was calculated from a calibration curve acquired from UV–vis spectrophotometer measurements based on the absorbance intensity at 480 nm, and the amount of cRGD peptide in HRED was measured by BCA assay according to the manufacturer's instruction. The following formulae were used to calculate encapsulation efficiency (EE) and loading efficiency (LE) of DOX: EE = (weight of loaded DOX)/(weight of initially added DOX) × 100%; LE = (weight of loaded DOX in EVs)/(weight of EVs) × 100%. In addition, the same method was used to load Taxol to obtain ET and HRET.

## Conflict of Interest

The authors declare no conflict of interest.

## Supporting information

Supporting Information

## Data Availability

Research data are not shared.
