# Supplementary material for: Lemon‐Derived Extracellular Vesicles Nanodrugs Enable to Efficiently Overcome Cancer Multidrug Resistance by Endocytosis‐Triggered Energy Dissipation and Energy Production Reduction
Source: Adv Sci (Weinh). 2022 Feb 20;9(20):2105274. doi: 10.1002/advs.202105274 (PMC9284146; doi:10.1002/advs.202105274)
Supplement: Supplementary file 1 — Supporting Information [file ADVS-9-2105274-s001.pdf]

ADVANCED SCIENCE
Open Access

Supporting Information

for *Adv. Sci.*, DOI 10.1002/advs.202105274

Lemon-Derived Extracellular Vesicles Nanodrugs Enable to Efficiently Overcome Cancer Multidrug Resistance by Endocytosis-Triggered Energy Dissipation and Energy Production Reduction

Qian Xiao, Wei Zhao, Chentian Wu, Xuejiao Wang, Jianping Chen, Xiubo Shi, Suinan Sha, Jinheng Li, Xiaomei Liang, Yulu Yang, Haoyan Guo, Ying Wang and Jun-Bing Fan**

ADVANCED SCIENCE
Open Access

Supporting Information

for *Adv. Sci.*, DOI: 10.1002/advs.202105274

Lemon-Derived Extracellular Vesicles Nanodrugs Enable to Efficiently Overcome Cancer Multidrug Resistance by Endocytosis-Triggered Energy Dissipation and Energy Production Reduction

Qian Xiao, Wei Zhao, Chentian Wu, Xuejiao Wang, Jianping Chen, Xiubo Shi, Suinan Sha, Jinheng Li, Xiaomei Liang, Yulu Yang, Haoyan Guo, Ying Wang and Jun-Bing Fan**

Supporting Information

Lemon-Derived Extracellular Vesicles Nanodrugs Enable to Efficiently Overcome Cancer Multidrug Resistance by Endocytosis-Triggered Energy Dissipation and Energy Production Reduction

Qian Xiao, Wei Zhao, Chentian Wu, Xuejiao Wang, Jianping Chen, Xiubo Shi, Suinan Sha,

Jinheng Li, Xiaomei Liang, Yulu Yang, Haoyan Guo, Ying Wang and Jun-Bing Fan**

Table of contents

1. Supplementary methods. 4
1.1 Materials and reagents. 4
1.2 Determination of the optimal ratio of EVs/HR in HRE. 5
1.3 Stability of EVs, ED, HRE and HRED. 5
1.4 In vitro drug release. 5
1.5 Complement activation assay. 6
1.6 Cell culture. 6
1.7 Western blotting. 7
1.8 Cellular uptake assay and intracellular localization. 7
1.9 Cytotoxicity assay *in vitro*. 8
1.10 Cell apoptosis and cell cycle analysis *in vitro*. 8
1.11 Mitochondrial membrane potential measurement 9
1.12 Animals and tumor models. 9
1.13 *In vivo* biodistribution. 10
1.14 Blood circulation time of ED and HRED. 10
1.15 Pharmacokinetics analysis *in vivo* 11
1.16 Tumor targeting ability *in vivo*. 11
1.17 Hemocompatibility detection. 11
1.18 *In vivo* biosafety assessment. 12
1.19 *In vivo* antitumor efficacy. 12
1.20 Histopathological Evaluation. 13
1.21 Intracellular accumulation of Rh123. 14
1.22 Immunofluorescence detection. 14
1.23 Endocytosis pathway detection. 15
1.24 RNA isolation and quantitative real-time PCR. 15
1.25 Intracellular ATP level detection. 16
1.26 Intracellular reactive oxygen species (ROS) level detection 16
1.27 Statistical analysis. 16
2. Supplementary figures. 18
Table S1. Characterization of HRE with different ratio of EVs/HR. 18
Table S2. Characterization of EVs-based nanodrugs. 18
Table S3. Loading weight in per μg EVs of HRED. 18
Table S4. Entrapment efficiency (EE, %) and loading efficiency (LE, %) of ED and HRED. 18
Table S5. IC50 of DOX, ED and HRED in different cells. 19
Table S6. IC50 of Taxol, ET and HRET in A2780/Taxol cells. 19
Table S7. Pharmacokinetics analysis of DOX after intraperitoneal injection of DOX, ED and HRED to rats at DOX dose of 10 mg/kg. 19

2.1 Characterization of HR........ 20
2.2 Cellular uptake of HRE with different ratio of EVs/HR in SKOV3/DOX cells........ 21
2.3 Identification of EVs markers........ 22
2.4 Characterization of HRED........ 23
2.5 DOX release from ED and HRED in PBS........ 24
2.6 Expression of integrin αv and β3 and resistance protein P-gp........ 25
2.7 Cellular uptake of EVs-based nanodrugs in SKOV3/DOX cells........ 26
2.8 Cellular uptake of EVs-based nanodrugs in SKOV3 cells........ 27
2.9 Cytotoxicity of EVs-based nanodrugs in SKOV3, SKOV3/Cisplatin and A2780/Taxol cells........ 28
2.10 Cell apoptosis in SKOV3/DOX cells treated with EVs-based nanodrugs........ 29
2.11 Cell cycle in SKOV3/DOX cells treated with EVs-based nanodrugs........ 30
2.12 Influence of EVs-based nanodrugs on mitochondrial membrane potential in SKOV3/DOX cells........ 31
2.13 *In vivo* biodistribution of EVs-based nanodrugs in orthotopic SKOV3-Luc ovarian cancer xenograft nude mouse model........ 32
2.14 Blood circulation time of EVs-based nanodrugs........ 33
2.15 Tumor targeting ability of EVs-based nanodrugs in orthotopic SKOV3-Luc ovarian cancer xenograft nude mouse model........ 34
2.16 Hemolysis test........ 35
2.17 Detection of liver and kidney functions of the C57BL/6 mice receiving treatments. 36
2.18 Antitumor efficacy of EVs in orthotopic SKOV3-Luc ovarian cancer xenograft nude mouse model........ 37
2.19 Antitumor efficacy of EVs-based nanodrugs in orthotopic SKOV3-Luc ovarian cancer xenograft nude mouse model........ 39
2.20 Systemic toxicity of EVs-based nanodrugs in orthotopic SKOV3-Luc ovarian cancer xenograft nude mouse model........ 42
2.21 Antitumor efficacy of EVs-based nanodrugs in orthotopic SKOV3/DOX-Luc ovarian cancer xenograft nude mouse model........ 43
2.22 Systemic toxicity of EVs-based nanodrugs in orthotopic SKOV3/DOX-Luc ovarian cancer xenograft nude mouse model........ 45
2.23 Influence of HR and DOX on protein expression in SKOV3/DOX cells........ 46
2.24 Localization of P-gp and EVs-based nanodrugs in SKOV3/DOX cells........ 47
2.26 Localization of CAV-1 and EVs-based nanodrugs in SKOV3/DOX cells........ 49

1. Supplementary methods.

1.1 Materials and reagents.

Heparin sodium salt (63007131, Mn = 1.25 kDa, 189 U/mg) was purchased from Sinopharm Chemical Reagent Co. (Shanghai, China). Doxorubicin (DOX, A603456) was purchased from Sangon Biotech (Shanghai, China), and cRGD peptide was synthesized by BAM BIOTECH CO., LTD (Xiamen, China). The BCA protein quantification assay kit (KGP903), Annexin V-APC apoptosis detection kit (KGA1021) and Cell Cycle detection kit (KGA512) were purchased from KeyGEN BioTECH (Nanjing, China). Alix (ab275377), TSG101 (ab125011), CD81 (ab109201), calnexin (ab22595), GAPDH (ab181602), Ki67 (ab21700), CD34 (ab81289), integrin αv (ab179475), integrin β3 (ab179473), caveolin-1 (CAV-1, ab192869), clathrin heavy chain (CHC, ab172958) primary antibodies and Alexa Fluor® 488 goat anti-rabbit secondary antibody (ab150081) were purchased from abcam company (USA). P-gp (22336-1-AP) primary antibody was purchased from Proteintech Group Inc, and horseradish peroxidase (HRP)-conjugated anti-rabbit secondary antibody (BS13278) was purchased from Bioworld Technology. PKH67 dye (PKH67GL) and PKH26 dye (PKH26GL) were obtained from Sigma-Aldrich. Hoechst 33342 (C1028), DAPI (C1002) and Mitochondrial membrane potential assay kit with JC-1 were purchased from Beyotime Biotechnology (Shanghai, China). Human complement fragment 3a (C3a) ELISA Kit (CSB-E08509h) was purchased from Cusabio Technology LLC (Wuhan, China). Chlorpromazine hydrochloride (CPZ, C834105-5g), methyl-β-cyclodextrin (MβCD, M812850-5g), amiloride hydrochloride (A832892-1g) and taxol (P875571-50mg) were purchased from Macklin Biochemical Co. Ltd (Shanghai, China). Rhodamine 123 (Rh123) was purchased from Aladdin Industrial Corporation (Shanghai, China). Sulfo-Cyanine3 NHS ester (R-H-3322) and Sulfo-Cyanine7 NHS ester (R-H-7109) were purchased from Xi'an ruixi Biological Technology Co. Ltd (Xi'an, China). Rat interferon α (IFN-α) ELISA Kit (HQ-30247), Rat interleukin 6 (IL-6) ELISA Kit

(HQ-30646), Rat interferon-inducible protein 10 (IP-10) ELISA Kit (HQ-30385), and Rat tumor necrosis factor α (TNF-α) ELISA Kit (HQ-30635) were purchased from Guanzhou Hongqing Bio-Tech Ltd (Guanzhou, China). Alanine aminotransferase (ALT) ELISA Kit (ml002169), aspartate aminotransferase (AST) ELISA Kit (ml058577), blood urea nitrogen (BUN) assay kit (ml076479) and creatinine (Cre) assay kit (ml959005) were purchased from Shanghai Enzyme-linked Biotechnology Co., Ltd (Shanghai, China). Cleaved caspase-3 (9661S) primary antibody was purchased from Cell Signaling Technology (USA).

1.2 Determination of the optimal ratio of EVs/HR in HRE.

In order to determine the optimal ratio of EVs/HR in HRE, we mixed and agitated 100 μg of EVs with different amounts of HR (2mg, 4mg and 6mg). Then, the obtained products were ultracentrifuged and marked as HRE1, HRE2 and HRE3, respectively. The size distribution, Zeta potential and polydispersity index (PDI) of ED, HRE and HRED were measured by dynamic light scattering (DLS) using a Zetasizer Nano-Zs (Malvern Instruments, UK). We also labeled HRE1, HRE2 and HRE3 with Cy3 and detected the cellular uptake in SKOV3/DOX cells by flow cytometry (Becton Dickinson, USA).

1.3 Stability of EVs, ED, HRE and HRED.

In this study, 10% FBS was added to the sample solution to simply mimic blood conditions. The size distribution and polydispersity index (PDI) of EVs, ED, HRE and HRED were detected by using DLS at different time. Detection of the different groups were taken in triplicate, and results were analyzed.

1.4 In vitro drug release.

DOX release profile from ED and HRED was determined by dialysis method in phosphate buffer (pH = 7.4) solutions. Briefly, ED and HRED (500 μg DOX content) were put into a dialysis bag (MWCO 3500 Da) and submerged fully into PBS (50 mL), then shaken at a

speed of 80 rpm at 37 °C. At the designated time intervals, 2 mL of samples were taken out for drug concentration measurement and replaced with equal volume of fresh PBS. DOX content in samples was measured by UV-vis spectrophotometer at 480nm. The drug release studies were performed in triplicate for each of the samples.

1.5 Complement activation assay.

To assess complement activation of samples in vitro, we detected the concentration of the activated complement fragment C3a by ELASA kits. Blood taken from volunteers by venous puncture was collected in vacuum tubes and put at room temperature for 30 min, and then plasma was obtained after centrifugation at 3500 rpm for 10 min. The test agent was incubated with 80 μL of plasma and 20 μL of EVs, ED, HR, HRE and HRED (EVs, ED, HRE and HRED contain the same amount of EVs at 10 μg, and the amount of HR in HRE is the same in HRED) at 37 °C for 2 h. The reaction was stopped and detected by a Multimode Reader (Biotek) at the wavelength of 450 nm. The experiment was performed in triplicate and repeated three times and the result displayed a representative of three independent experiments.

1.6 Cell culture.

The human DOX-sensitive ovarian cancer cells SKOV3 and human Taxol-sensitive ovarian cancer cells A2780 were obtained from the Cell Bank of Type Culture Collection of the Chinese Academy of Sciences (Shanghai, China). The DOX-resistant ovarian cancer cells SKOV3/DOX and Cisplatin-resistant ovarian cancer cells SKOV3/Cisplatin were constructed by inducing SKOV3 with DOX and Cisplatin, and the Taxol-resistant ovarian cancer cells A2780/Taxol was constructed by inducing A2780 with Taxol in our laboratory. The SKOV3-Luc and SKOV3/DOX-Luc cells were generated in our laboratory via transfection with a reporter gene encoding firefly luciferase. A2780 and A2780/Taxol were cultured in DMEM,

and the other cells were cultured in RPMI-1640 medium, supplemented with 10% FBS (Gibco, USA) and 1% penicillin-streptomycin (Gibco, USA). All cells were maintained in a humidified incubator with 5% CO_2 at 37 °C.

1.7 Western blotting.

Cells, lemon juice, 300 μg of EVs and 300 μg of HRE were lysed with 100 μL RIPA buffer containing protease inhibitor (Beyotime Biotechnology, China). SKOV3/DOX cells were seeded at a density of 2×10^5 cells/well in 6-well plates and incubated at 37 °C for 12 h. After treating with free DOX, ED and HRED (10 μg/mL DOX), and EVs and HRE (equivalently to HRED), and HR (equivalently to HRE) for 24 h, the cells were harvested, and lysed on ice. The concentration of total proteins was determined using BCA protein quantification assay kit. Equal quantities of protein were separated by 5-10% SDS-PAGE and transferred onto PVDF membranes (Millipore, USA). The PVDF membranes were blocked for 1 h and incubated with primary antibody overnight at 4 °C, followed by incubation with HRP-conjugated anti-rabbit secondary antibodies for 1 h. The signals were detected using a Bio-Rad ChemiDoc™ MP Imaging System (Bio-Rad, USA). Western blottings were quantified using Image Lab software.

1.8 Cellular uptake assay and intracellular localization.

SKOV3/DOX and SKOV3 cells were seeded in 6-well plates at a density of 4×10^5 cells/well and incubated for 12 h. Then PKH67-labeled EVs and HRE (with EVs concentration 2 μg/mL), free DOX, ED and HRED (with DOX concentration 2.5 μg/mL) were added to the well and incubated for 4 h at 37 °C, respectively. After incubation, cells were washed with cold PBS, centrifuged and resuspended in 0.3 mL PBS solution for detection of DOX (PE channel) or PKH67 signal (FITC channel) by flow cytometry (Becton Dickinson, USA). The results were analyzed using FlowJo 7.6 software.

Confocal fluorescence microscopy was used to detect intracellular localization of samples. SKOV3/DOX and SKOV3 cells were seeded on the cover glasses in 6-well plates at a density of 2×10^5 cells/well and incubated for 24 h. Then free DOX, PKH67-labeled ED and HRED (with DOX concentration 2.5 μg/mL) were added and incubated with SKOV3/DOX and SKOV3 cells for 4 h. After washed with PBS, the cells were fixed with 4% paraformaldehyde for 10 min, followed by staining with Hoechst 33342 (Beyotime, China). The intracellular localization of EVs, HRE and DOX in different groups was visualized using a confocal microscope (Nikon, Japan).

1.9 Cytotoxicity assay *in vitro*.

SKOV3/DOX, SKOV3, SKOV3/Cisplatin and A2780/Taxol cells were seeded in a 96-well plates at an initial density of 3×10^3 cells/well. After incubation for 12 h, the culture medium of each well was replaced with 200 μL of fresh medium containing different concentrations of free DOX, ED and HRED. In addition, A2780/Taxol cells also incubated with Taxol, ET and HRET. After 48 h, the medium was replaced with 110 μL of fresh solution (100 μL medium plus 10 μL of MTT solution). After 4 h of incubation, the medium was removed and the wells were added to 150 μL DMSO to dissolve the crystals formed by living cells, and then the absorbance was measured at 490 nm using a microplate reader (Synergy2, Bio-Tek, USA). Cell viability in each group was calculated as a percentage of the absorbance to that of the control experiment.

1.10 Cell apoptosis and cell cycle analysis *in vitro*.

SKOV3/DOX cells were seeded in 6-well plates at an initial density of 3×10^5 cells/well, and then incubated with free DOX, ED and HRED (5 μg/mL DOX), and EVs and HRE (equivalently to HRED), and HR (equivalently to HRE) for 24 h. At the end of the incubation, the cells were trypsinized and washed with cold PBS. For cell apoptosis analysis, the cells

were stained using Annexin V-APC apoptosis detection kit (KeyGEN BioTECH, China) and DAPI (Beyotime, China) according to the manufacturer's protocol, and then analyzed by flow cytometry. For determination of the cell cycle, the cells were fixed in 70% precooled ethanol at 4 °C overnight, then washed with PBS and stained using Cell Cycle Detection Kit (KeyGEN BioTECH, China) for 30 min in the dark at room temperature, and finally analyzed for DNA content by flow cytometry.

1.11 Mitochondrial membrane potential measurement

SKOV3/DOX cells were seeded in a 6-well plates at an initial density of 3×10^5 cells/well, and then incubated with free DOX, ED and HRED (2.5 μg/mL DOX), and EVs and HRE (equivalently to HRED), and HR (equivalently to HRE) for 24 h. At the end of the incubation, the cells were trypsinized, washed and stained using a Mitochondrial membrane potential assay kit with JC-1 (Beyotime, China) for 30 min according to the manufacturer's protocol. Normal mitochondria with a higher potential were labeled with red fluorescence, while unhealthy mitochondria with a lower potential were labeled with green fluorescence. The fluorescence signals were analyzed by flow cytometry and then red: green ratio was calculated after the flow cytometry assay.

1.12 Animals and tumor models.

Female BALB/c nude mice, C57BL/6 mice aged 6-7 weeks (15-20 g) and adult Sprague-Dawley (SD) rats (180-200 g) were purchased from Guangdong Medical Laboratory Animal Center. All procedures for animal use were approved by the Institutional Animal Care and Use Committee (IACUC) at Southern Medical University, Guangzhou, China. The nude mice were fasted for 12 h before operation but with free access to water. They were anesthetized by intraperitoneally injecting 1% sodium pentobarbital (8 μL/g body weight). The anesthetized animals were placed in prone position and fixed on an operating table. An approximately 1 cm of incision was made vertically near the spine between the right costal arch and the femur.

The abdominal cavity was opened according to an anatomical level to expose the ipsilateral ovary and the fallopian tube. The ovary was carefully fixed using ophthalmic forceps, and the prepared SKOV3/DOX-Luc or SKOV3-Luc cells suspension (10 μL, 4 × 10^4 cells/μL) was gradually injected into the ovary. After verifying the absence of active bleeding, we carefully put back the ovary to the abdominal cavity and sutured the incision. The postoperative mice were fed to their cages. After surgery, the growth of tumors was monitored by bioluminescence imaging using an *in vivo* imaging system (IVIS Lumina II, Caliper, USA).

1.13 *In vivo* biodistribution.

The orthotopic SKOV3/DOX-Luc or SKOV3-Luc ovarian cancer xenograft nude mouse model (n = 3) were intraperitoneally injected with free Cy7, Cy7-labeled ED and HRED (Cy7-ED and Cy7-HRED). At 0.5, 3, 6, 12, 24, 48, 72, and 96 h after injection, the mice were anesthetized, and the Cy7 fluorescence and luciferase signal (96 h) imagings were visualized by IVIS. The mice were sacrificed at 96 h and the ovarian primary tumors and major organs (heart, liver, spleen, lung, and kidney) were harvested and visualized by IVIS to compare relative accumulation.

1.14 Blood circulation time of ED and HRED.

To evaluate the stability of circulating ED and HRED *in vivo*, free Cy7, Cy7-ED and Cy7-HRED (Cy7 dosage, 50 μg/kg) were slowly injected intraperitoneally into adult SD rats (n = 3). After injection, the blood was collected from the SD rats at different time points (0.5 h, 3 h, 6 h, 12 h, 24 h, 48 h, 72 h, 96 h, 120 h and 144 h) and detected using IVIS immediately. Meanwhile, the blood cells and plasma were separated by centrifuging at 3000 rpm for 15 min, and then the Cy7 signal in blood cells and plasma was also detected. The signal from rats without any treatment was used as the control.

1.15 Pharmacokinetics analysis *in vivo*

The SD rats (180-200 g) were randomly divided into three groups (n = 3), and free DOX, ED and HRED (10 mg/kg DOX) were slowly injected intraperitoneally to evaluate the pharmacokinetics *in vivo*. After injection, the blood was collected from the SD rats at different time points (0.167 h, 0.5 h, 1 h, 3 h, 6 h, 12 h, 24 h, 48 h, 72 h, 96 h) and the plasma was harvested by centrifuging at 3000 rpm for 15 min, and then the DOX were extracted from plasma by deproteinization using acetonitrile, followed by centrifugation at 10,000 rpm for 10 min. Then the supernatant was loaded onto a black 96-well plate for fluorescence determination (Ex 480 nm/Em 590 nm) by cell imaging multi-mode reader (BioTek Cytation 5, USA) and the concentration of DOX was calculated by comparing the fluorescence with a calibration curve generated by spiking known amounts of DOX into rat plasma. The pharmacokinetic results were analyzed by DAS2 software (China).

1.16 Tumor targeting ability *in vivo*.

The orthotopic SKOV3/DOX-Luc or SKOV3-Luc ovarian cancer xenograft nude mouse model (n = 3) were intraperitoneally injected with DOX, ED and HRED (2.5 mg/kg DOX). After 24 h post administration, the mice were sacrificed to collect the tumors. Then, the tumor tissues of each group were fixed in 4% paraformaldehyde for 24 h, embedded in paraffin wax, cut into 4 μm-thick sections, and stained with Hoechst 33342 for 5 min at room temperature. After washing three times with PBS, the sections were immediately examined under confocal microscope.

1.17 Hemocompatibility detection.

To investigate the hemocompatibility of EVs and HRE in vitro, hemolysis test was performed. Red blood cells were collected from healthy SD rats and incubated with various concentrations of EVs and HRE in tubes at room temperature for 3 h. Subsequently, the samples were centrifuged at 4000 rpm for 10 min and photographed, and the absorbance of

supernatant at 541 nm was measured using a microplate reader (Synergy2, Bio-Tek, USA). Red blood cells were incubated with either PBS or ultrapure water to use as a negative (-) or postivie control (+), respectively.

1.18 *In vivo* biosafety assessment.

To investigate whether different samples induced the changes of inflammatory cytokine levels *in vivo*, we assessed the concentrations of IL-6, IFN-α, TNF-α and IP-10 in serum from adult SD rats after intraperitoneal administration of different samples. Briefly, the SD rats were divided into seven groups (n = 3), and intraperitoneally injected with free DOX, ED and HRED (2.5 mg/kg DOX), and EVs and HRE (equivalently to HRED), and HR (equivalently to HRE), using PBS as control. At 24 h after administration, the SD rats were anesthetized and the blood was dripped into a tube. After clotting for 2 h at room temperature, the blood cells and serum were separated by centrifuging at 3000 rpm for 15 min. The concentrations of each cytokine in the serum were determined using commercially available ELISA kits according to the manufacturer's instruction.

To investigate the systemic toxicity of EVs, DOX, ED and HRED *in vivo*, the serum from the blood samples of the C57BL/6 mice were collected for the measurement of biochemical analyses. Briefly, the C57BL/6 mice were divided into five groups (n = 3), and intraperitoneally injected with DOX, ED and HRED (2.5 mg/kg DOX), and EVs (equivalently to HRED), using PBS as control. At 24 h after administration, the blood of C57BL/6 mice was collected. After clotting for 2 h at room temperature, the blood cells and serum were separated by centrifuging at 3000 rpm for 15 min. The concentrations of alanine aminotransferase (ALT), aspartate aminotransferase (AST), blood urea nitrogen (BUN) and creatinine (Cre) in the serum were determined according to the manufacturer's instruction.

1.19 *In vivo* antitumor efficacy.

Three days after injecting SKOV3/DOX-Luc or SKOV3-Luc cells into the ovaries, the tumors were detected by IVIS luminescent imaging and recorded. Consequently, orthotopic SKOV3/DOX-Luc ovarian cancer xenograft nude mouse model were randomly divided into five different groups (n = 6), and then the mice were intraperitoneally injected to PBS, EVs (the equivalent dose with HRED), free DOX, ED or HRED (2.5 mg/kg DOX) every three days. The body weight was detected every 4 days, and the tumor size was quantified by IVIS every 4 days. After 20 days of treatment, mice were sacrificed and the ovarian tumors and major organs (heart, liver, spleen, lung, kidney, and intestine) were excised and detected for bioluminescence signals. The weights and volumes of ovarian tumors and the formation of ascites were analyzed. Tumor volume was calculated as: V = larger diameter × (smaller diameter)^2/2.

For the orthotopic SKOV3-Luc ovarian cancer xenograft nude mouse model, mice were randomly divided into four different groups (n = 5), and the treatment conditions were as follows: PBS, DOX, ED or HRED (2.5 mg/kg DOX) were administered intraperitoneally every three days. The body weight and tumor size were monitored every 4 days. After 16 days of treatment, mice were sacrificed and the ovarian primary tumors, metastatic nodules and major organs were excised and detected for bioluminescence signals. The weights and volumes of ovarian primary tumors and metastasis nodules were detected. In addition, the antitumor efficacy of EVs was also evaluated in the orthotopic SKOV3-Luc ovarian cancer xenograft nude mouse model using the same protocol.

1.20 Histopathological Evaluation.

The organs (heart, liver, spleen, lung, kidney, and intestine) and tumors collected from different treatment groups were fixed with 4% paraformaldehyde for 48 h at room temperature. Then, all tissues were embedded in paraffin wax, cut into 4-μm-thick sections, stained with hematoxylin and eosin (H&E) or used for immunohistochemical (IHC) analysis

of Cleaved caspase-3, Ki67 and CD34 expression according to standard clinical laboratory protocols. For IHC assay, antigen retrieval was done with 10 mM citrate buffer (pH 6.0), and endogenous peroxidase was blocked by 3% hydrogen peroxide solution. Detection was done with DAB working solution (GeneTech, China), which results in brown color staining. The nuclei were counter-stained with haematoxylin. Microscopic images of the tissues were acquired using an optical microscope (Nikon, Japan).

1.21 Intracellular accumulation of Rh123.

SKOV3/DOX cells were seeded at a density of 2×10^5 cells/well in 6-well plates and incubated for 12 h. Subsequently, the cells were treated with free DOX, ED and HRED (2.5 μg/mL DOX), and EVs and HRE (equivalently to HRED), and HR (equivalently to HRE) for 24 h. Next, the cells were washed and cultured in the media containing Rh123 (1 μg/mL) for 30 min, and then the cells were collected and analyzed by flow cytometry. The intracellular accumulation of Rh123 was analyzed as the percentage of that Rh123 uptake in control.

1.22 Immunofluorescence detection.

Cover glasses were placed at the bottom in 6-well plates. SKOV3/DOX cells were cultured on the cover glasses in the plates at a density of 2×10^5 cells/well at 37 °C for 24 h. Then the medium was replaced with 2 mL of fresh medium containing PKH26-EVs and PKH26-HRE (2 μg/mL EVs), ED and HRED (2.5 μg/mL DOX) and incubated at 37 °C for 2 h. After incubation, cells were fixed with 4% paraformaldehyde for 10 min at room temperature. After washing, cells were permeabilized using 0.25% Triton X-100 in PBS for 15 min and blocked for 30 min in 1% BSA at room temperature. After blocking, cells were incubated with P-gp or CAV-1 antibodies overnight at 4 °C. Unbounded antibody was removed by washing with PBS for three times. Cells were then incubated with Alexa Fluor® 488 goat anti-rabbit secondary antibody at room temperature for 1 h, and washed three times with PBS. Then cells were

stained with Hoechst 33342 for 5 min, and washed three times with PBS. The samples were examined by confocal microscope.

1.23 Endocytosis pathway detection.

SKOV3/DOX cells were cultured in the 6-well plates at a density of 2 × 10^5 cells/well at 37 °C. After 12 h, cells were pre-incubated at 4°C for 1 h and then the PKH67-EVs and PKH67-HRE (with EVs concentration 2 μg/mL), ED and HRED (with DOX concentration 2.5 μg/mL) were added and incubated for another 2 h at 4 °C. In other treatment groups, cells were pretreated for 1 h at 37 °C with the following pharmacological inhibitors at their indicated concentrations: sodium azide (NaN_3) (1 mg/mL), chlorpromazine (CPZ) (10 μg/mL), methyl-β-cyclodextrin (MβCD) (2.5 mM) and amiloride (15.1 μg/mL), and then the PKH67-EVs, PKH67-HRE, ED and HRED were added and incubated for another 2 h at 37 °C. Next, the cells were washed with cold PBS solution, centrifuged and resuspended in PBS solution. After these, the PKH67 and DOX signals were detected by flow cytometry. The inhibition rate was analyzed as the percentage of that internalized in control.

1.24 RNA isolation and quantitative real-time PCR.

The mRNA level of CAV-1 and clathrin heavy chain (CHC) were tested by quantitative PCR (q-PCR). SKOV3/DOX cells were seeded at a density of 5 × 10^5 cells/well in 6-well plates and incubated for 24 h. Next, the cells were treated with free DOX, ED and HRED (10 μg/mL DOX), and EVs and HRE (equivalently to HRED), and HR (equivalently to HRE) for 6 h. Total RNA for each sample was extracted using TRIzol reagent (Invitrogen, USA), quantified using a NanoDrop One spectrophotometer (Thermo Fisher Scientific, USA) and reverse-transcribed into cDNA using the PrimeScript RT Reagent Kit (Takara, Japan) according to the manufacturer's directions. Quantitative real-time PCR was performed on a Mx3005p Thermal Cycler (Agilent Technologies, USA) with SYBR Premix Ex Taq II (Takara, Japan).

Independent experiments were conducted in triplicate, and GAPDH served as an internal control. The following primers sequences (Ruibiotech, Inc., Beijing, China) were used: CAV-1, forward 5'-GCG ACC CTA AAC ACC TCA AC-3' and reverse 5'-ATG CCG TCA AAA CTG TGT GTC-3'; clathrin heavy chain (CHC), forward 5'-TGA TCG CCA TTC TAG CCT TGC-3' and reverse 5'-CTC CCA CCA CAC GAT TTT GCT-3'; and GAPDH, forward 5'-GGA GCG AGA TCC CTC CAA AAT-3' and reverse 5'- GGC TGT TGT CAT ACT TCT CAT GG-3'.

1.25 Intracellular ATP level detection.

The intracellular ATP level was assayed using a luciferase-based ATP assay kit (Beyotime, China). SKOV3/DOX cells at a density of 2×10^5 cells/well were seeded in 6-well plates, and then incubated with free DOX, ED and HRED (2.5 μg/mL DOX), and EVs and HRE (equivalently to HRED), and HR (equivalently to HRE) for 24 h. According to the instruction, cells were lysed and centrifuged at 12,000×g for 5 min. The supernatant was collected and mixed with the ATP detection working dilution in a ratio of 1:4. The luminescence intensity was detected by cell imaging multi-mode reader (BioTek Cytation 5, USA).

1.26 Intracellular reactive oxygen species (ROS) level detection .

SKOV3/DOX cells at a density of 2×10^5 cells/well were seeded in 6-well plates, and then incubated with free DOX, ED and HRED (2.5 μg/mL DOX), and EVs and HRE (equivalently to HRED), and HR (equivalently to HRE) for 24 h. Next, the cells were washed and cultured in the FBS free media containing 10 μM DCFH-DA (Beyotime, China) for 30 min, and then the cells were collected and analyzed by flow cytometry.

1.27 Statistical analysis.

All data were reported as mean ± SD. One-way ANOVA or unpaired two-tailed Student's t-tests was used to establish statistical significance using IBM SPSS 19.0 software. Differences were considered significance at *P < 0.05, **P < 0.01, ***P < 0.001.

2. Supplementary figures.

Table S1. Characterization of HRE with different ratio of EVs/HR.

Samples	Size (nm)	Zeta (mV)	PDI
HRE1	176.03 ± 0.98	-30.33 ± 1.70	0.23 ± 0.03
HRE2	202.70 ± 5.07	-31.43 ± 1.16	0.25 ± 0.01
HRE3	354.87 ± 9.33	-33.63 ± 1.30	0.32 ± 0.01

Data were presented as means ± SD, n = 3.

Table S2. Characterization of EVs-based nanodrugs.

Samples	Size (nm)	Zeta (mV)	PDI
EVs	151.63 ± 5.20	-29.63 ± 0.68	0.24 ± 0.006
ED	155.80 ± 12.71	-25.73 ± 0.32	0.17 ± 0.003
HRE	202.70 ± 5.07	-31.43 ± 1.16	0.25 ± 0.014
HRED	198.20 ± 6.21	-29.70 ± 0.95	0.16 ± 0.007

Data were presented as means ± SD, n = 3.

Table S3. Loading weight in per μg EVs of HRED.

Loading weight (μg in per μg of EVs)	cRGD peptide	DOX
EHRD	0.44 ± 0.01	1.35 ± 0.04

Data were presented as means ± SD, n = 3.

Table S4. Entrapment efficiency (EE, %) and loading efficiency (LE, %) of ED and HRED.

	ED	HRED
Entrapment Efficiency (EE, %)	23.97 ± 2.26	33.65 ± 2.76

Loading Efficiency (LE, %)	16.74 ± 1.20	18.84 ± 0.56

Data were presented as means ± SD, n = 3.

Table S5. IC50 of DOX, ED and HRED in different cells.

Treatments	Cell lines / IC50 ± SD (μg/mL)			
	SKOV3/DOX	SKOV3	SKOV3/Cisplatin	A2780/Taxol
DOX	5.55 ± 0.03	0.37 ± 0.05	0.81 ± 0.03	0.91 ± 0.02
ED	4.42 ± 0.03	0.15 ± 0.02	0.55 ± 0.02	0.39 ± 0.02
HRED	2.30 ± 0.02	0.11 ± 0.03	0.17 ± 0.02	0.25 ± 0.04

Data were presented as means ± SD, n = 5.

Table S6. IC50 of Taxol, ET and HRET in A2780/Taxol cells.

Treatments	Cell lines / IC50 ± SD (μg/mL)
	A2780/Taxol
Taxol	1.20 ± 0.07
ET	0.82 ± 0.03
HRET	0.64 ± 0.03

Data were presented as means ± SD, n = 5.

Table S7. Pharmacokinetics analysis of DOX after intraperitoneal injection of DOX, ED and HRED to rats at DOX dose of 10 mg/kg.

	C_{max}(ng/mL)	CL(mL/(h/kg))	AUC (ng/mL*h)	MRT (h)	$t_{1/2}$ (h)
DOX	561.5 ± 72.7	7 ± 3.4	1310.9 ± 28	2.4 ± 1.4	1.9 ± 0.04
ED	1264.0 ± 6.7	-	7800.1 ± 77	9.2 ± 3.6	6.7 ± 0.1
HRED	2266.1 ± 31.1	-	106079.2 ± 85	34.2 ± 3.4	24 ± 0.3

C_{max}: Maximum concentration; CL: Clearance; AUC: Area under the curve; MRT: Mean resi dence time; $t_{1/2}$: Half-life in rat plasma. Data were presented as means ± SD, n = 3.

2.1 Characterization of HR.

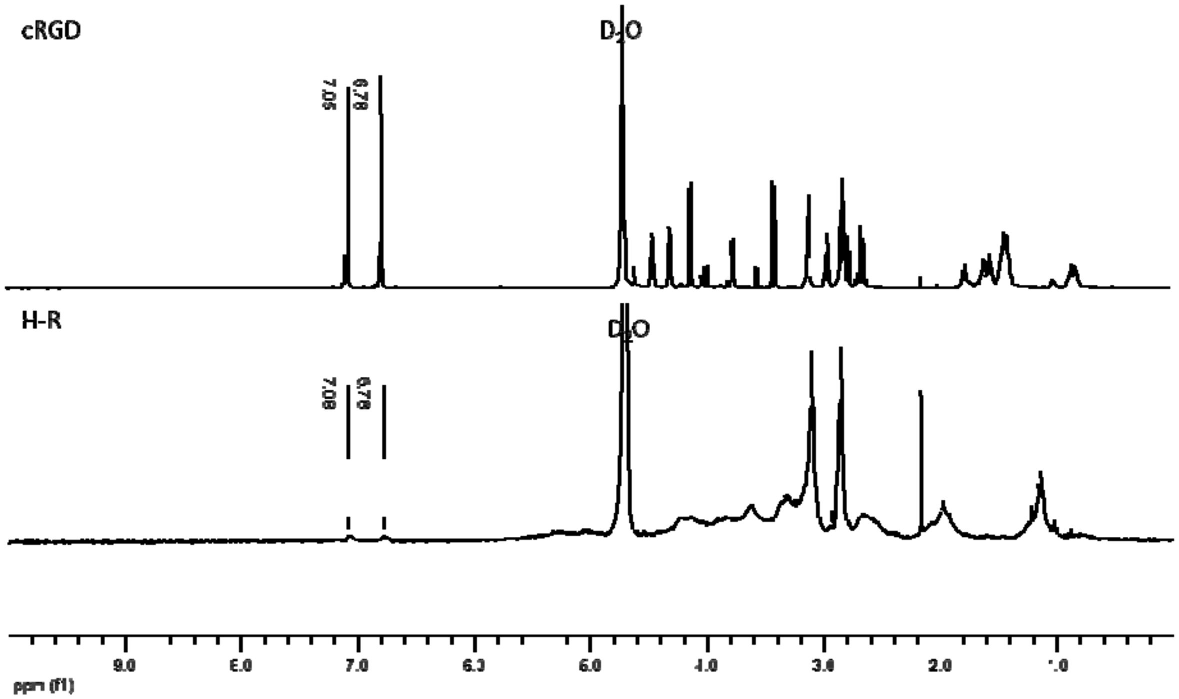

Figure S1. ^{1}H NMR spectra of cRGD and HR in D_2O.

2.2 Cellular uptake of HRE with different ratio of EVs/HR in SKOV3/DOX cells.

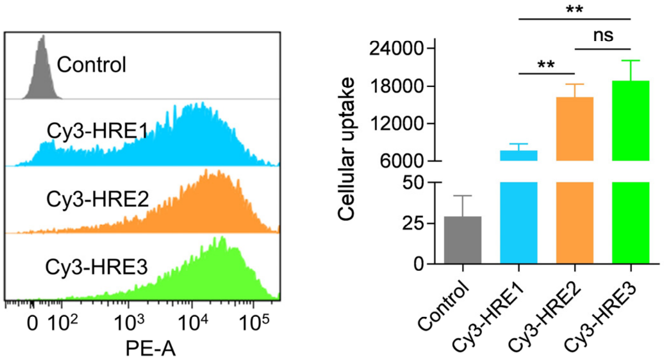

Figure S2. The cellular uptake of Cy3-HRE1, Cy3-HRE2 and Cy3-HRE3 (2 µg/mL EVs) in SKOV3/DOX cells, as detected by flow cytometry. Results are represented as mean ± SD (n = 3). *$P < 0.05$, **$P < 0.01$, and ***$P < 0.001$.

2.3 Identification of EVs markers.

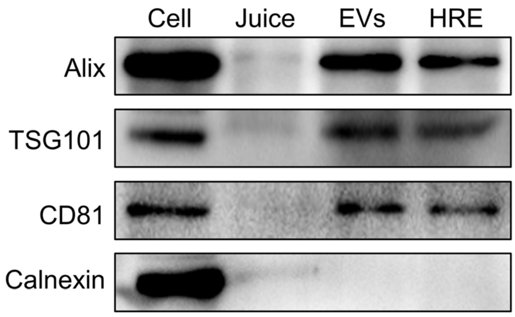

Figure S3. The expression of Alix, TSG101, CD81 and calnexin of lemon juice, lemon EVs and HRE as measured by western blotting using SKOV3/DOX cell lysates as positive control.

2.4 Characterization of HRED.

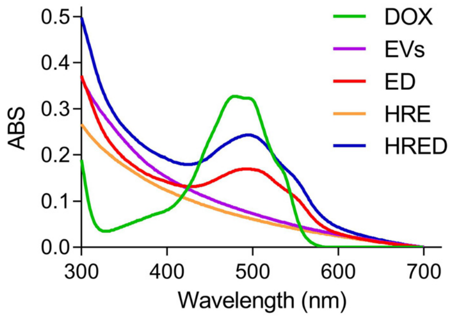

Figure S4. UV-vis spectra of DOX, lemon-derived EVs, ED, HRE and HRED in water.

2.5 DOX release from ED and HRED in PBS.

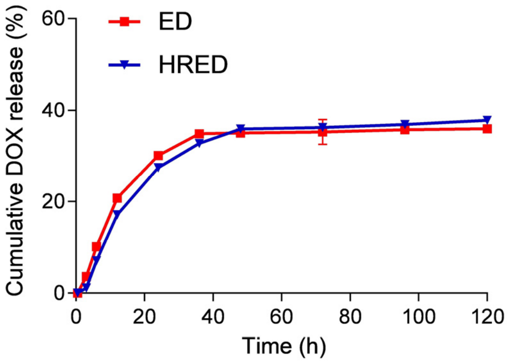

Figure S5. The cumulative release of DOX from ED and HRED in normal physiological condition (PBS, pH 7.4).

2.6 Expression of integrin αv and β3 and resistance protein P-gp.

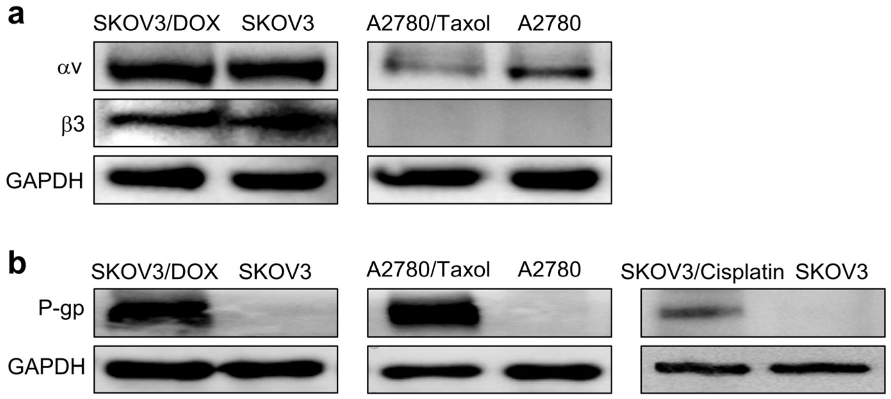

Figure S6. a) Western blotting analysis of integrin αv and β3 in SKOV3/DOX, SKOV3, A2780/Taxol and A2780 cells. b) Western blotting analysis of P-gp in SKOV3/DOX, SKOV3, SKOV3/Cisplatin, A2780/Taxol and A2780 cells.

2.7 Cellular uptake of EVs-based nanodrugs in SKOV3/DOX cells.

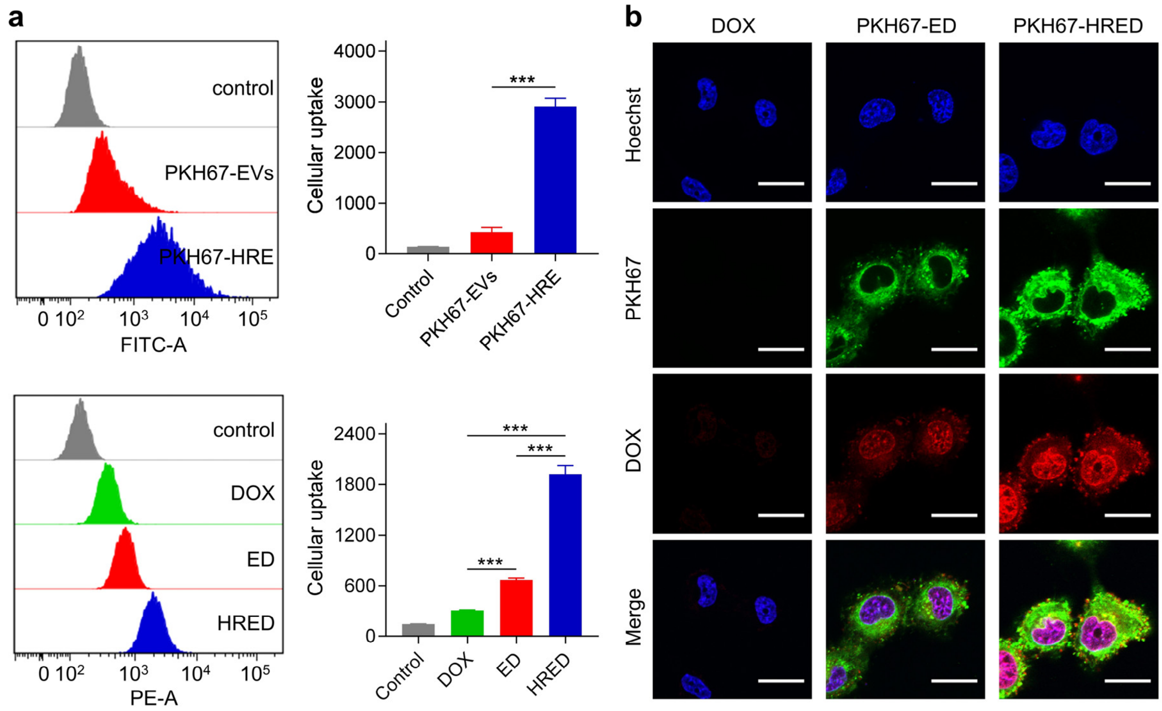

Figure S7. a) The cellular uptake of PKH67-EVs, PKH67-HRE (2 μg/mL EVs), DOX, ED and HRED (2.5 μg/mL DOX) in SKOV3/DOX cells, as detected by flow cytometry. b) The intracellular localization of DOX, PKH67-ED and PKH67-HRED (2.5 μg/mL DOX) in SKOV3/DOX cells as detected by confocal microscope. Scale bar: 20 μm. Results are represented as mean ± SD (n = 3). **P* < 0.05, ***P* < 0.01, and ****P* < 0.001.

2.8 Cellular uptake of EVs-based nanodrugs in SKOV3 cells.

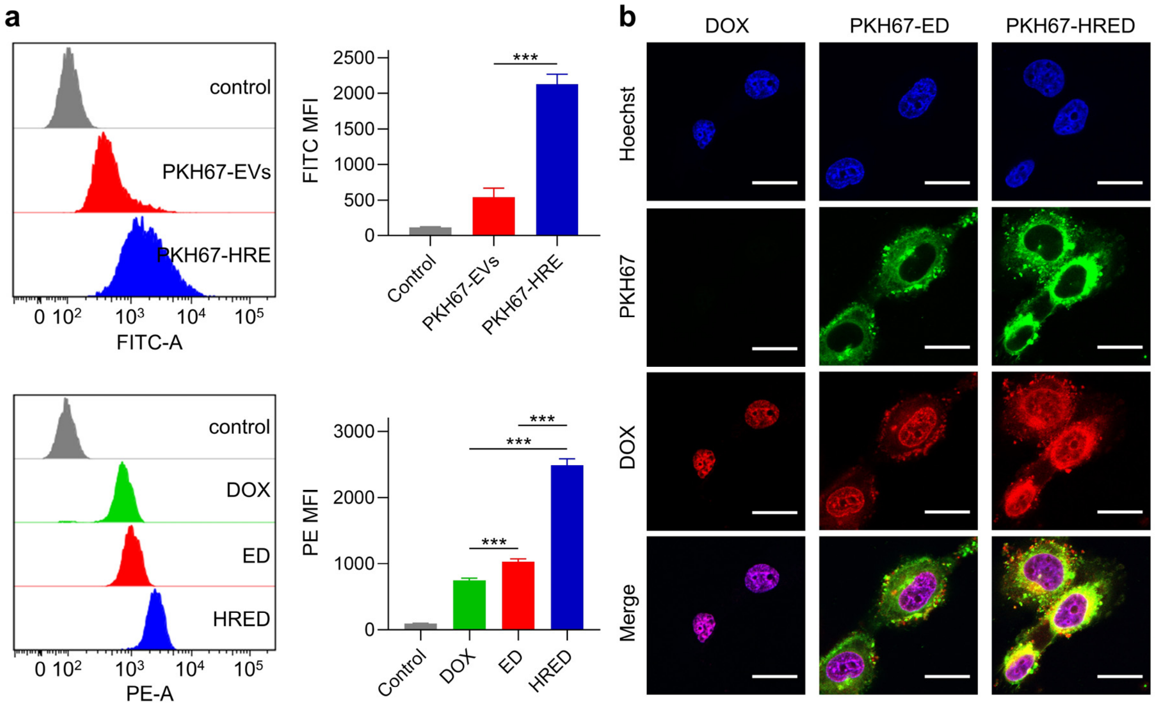

Figure S8. a) Cellular uptake of PKH67-EVs, PKH67-HRE (2 μg/mL EVs), DOX, ED and HRED (2.5 μg/mL DOX) in SKOV3 cells for 4 h, as detected by flow cytometry. b) Intracellular localization of DOX, PKH67-ED and PKH67-HRED (2.5 μg/mL DOX) in SKOV3 cells for 4 h, as detected by confocal microscope. Scale bar: 20 μm. Results are represented as mean ± SD (n = 3). $*P < 0.05$, $**P < 0.01$, and $***P < 0.001$.

2.9 Cytotoxicity of EVs-based nanodrugs in SKOV3, SKOV3/Cisplatin and A2780/Taxol cells.

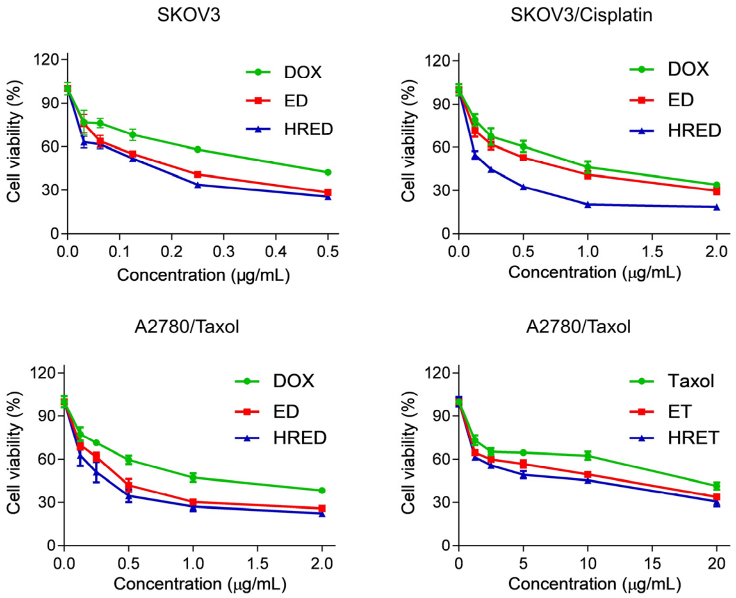

Figure S9. Cell viability of SKOV3, SKOV3/Cisplatin and A2780/Taxol cells treated with DOX, ED and HRED for 48 h, and cell viability of A2780/Taxol cells treated with Taxol, ET and HRET for 48 h.

2.10 Cell apoptosis in SKOV3/DOX cells treated with EVs-based nanodrugs.

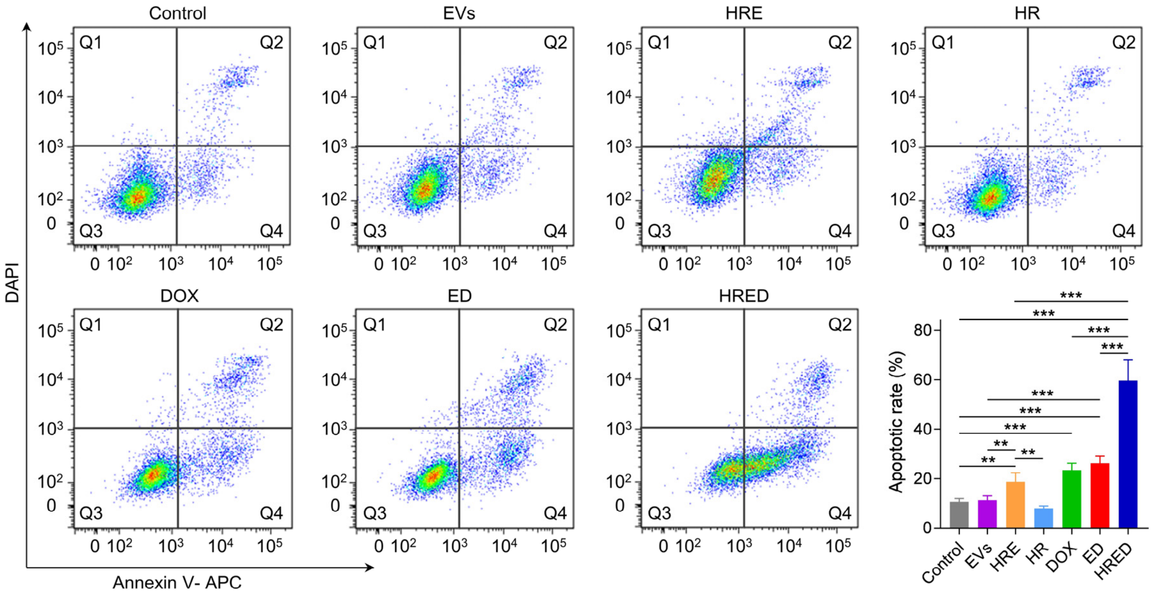

Figure S10. The flow cytometry results of cell apoptosis of SKOV3/DOX cells treated with free DOX, ED and HRED (5 μg/mL DOX), and EVs and HRE (equivalently to HRED), and HR (equivalently to HRE) for 24 h. Q1, necrotic cells; Q2, late apoptotic cells; Q3, live cells; Q4, early apoptotic cells. Results are represented as mean ± SD (n = 3). *$P < 0.05$, **$P < 0.01$, and ***$P < 0.001$.

2.11 Cell cycle in SKOV3/DOX cells treated with EVs-based nanodrugs.

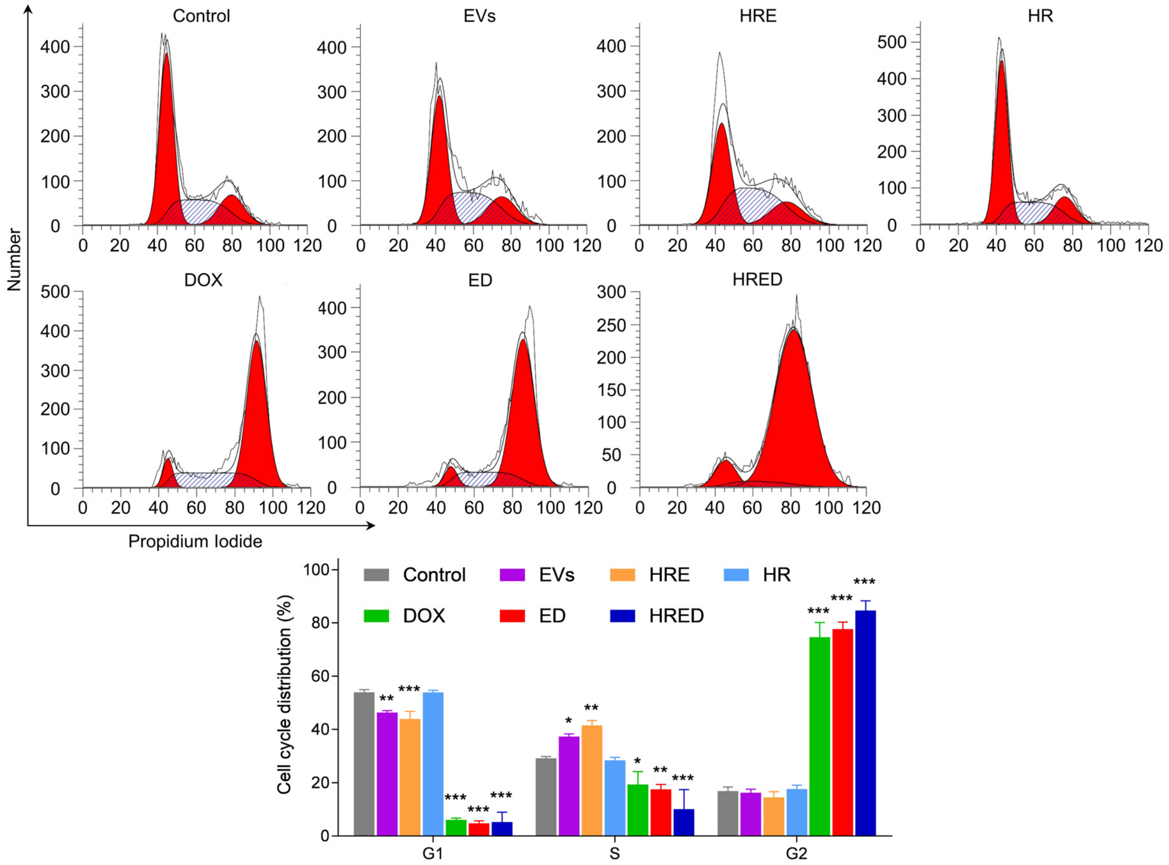

Figure S11. The flow cytometry results of cell cycle distribution of SKOV3/DOX cells treated with free DOX, ED and HRED (5 μg/mL DOX), and EVs and HRE (equivalently to HRED), and HR (equivalently to HRE) for 24 h. Results are represented as mean ± SD (n = 3). **P* < 0.05, ***P* < 0.01, and ****P* < 0.001.

2.12 Influence of EVs-based nanodrugs on mitochondrial membrane potential in SKOV3/DOX cells.

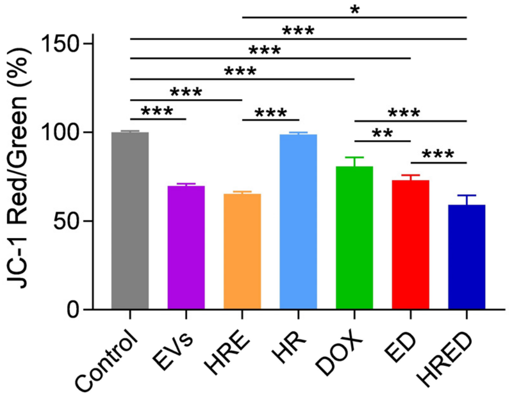

Figure S12. The flow cytometry results of the change of mitochondrial membrane potential in SKOV3/DOX cells treated with free DOX, ED and HRED (2.5 μg/mL DOX), and EVs and HRE (equivalently to HRED), and HR (equivalently to HRE) for 24 h. Normal mitochondria with a higher potential were labeled with red fluorescence, while unhealthy mitochondria with a lower potential were labeled with green fluorescence. Results are represented as mean ± SD (n = 3). *$P < 0.05$, **$P < 0.01$, and ***$P < 0.001$.

2.13 *In vivo* biodistribution of EVs-based nanodrugs in orthotopic SKOV3-Luc ovarian cancer xenograft nude mouse model.

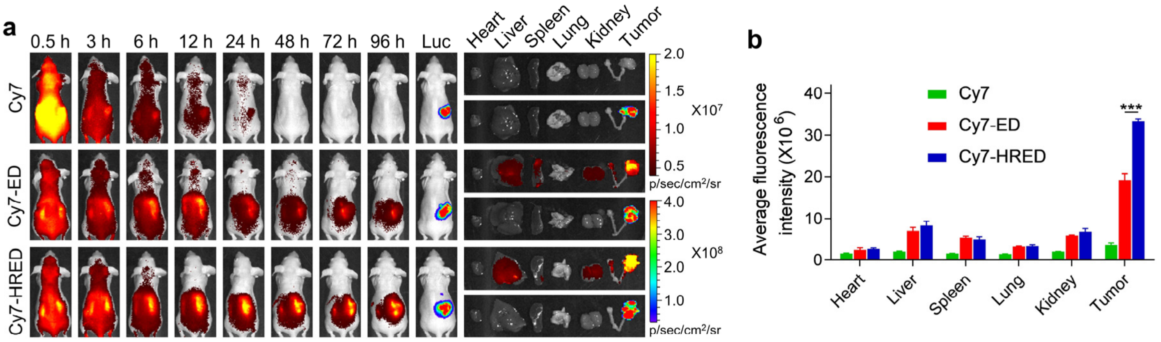

Figure S13. a) *In vivo* fluorescence images of orthotopic SKOV3-Luc ovarian cancer xenograft nude mouse model at different time points after intraperitoneal injection of free Cy7, Cy7-ED and Cy7-HRED (containing with 50 µg/kg Cy7). After 96 h, mice were sacrificed and their major organs and tumors were collected to detect Cy7 and Luc signals. b) The corresponding semiquantitative results of ex vivo organs and tumors in different groups. Results are represented as mean ± SD (n = 3). **P* < 0.05, ***P* < 0.01, and ****P* < 0.001.

2.14 Blood circulation time of EVs-based nanodrugs.

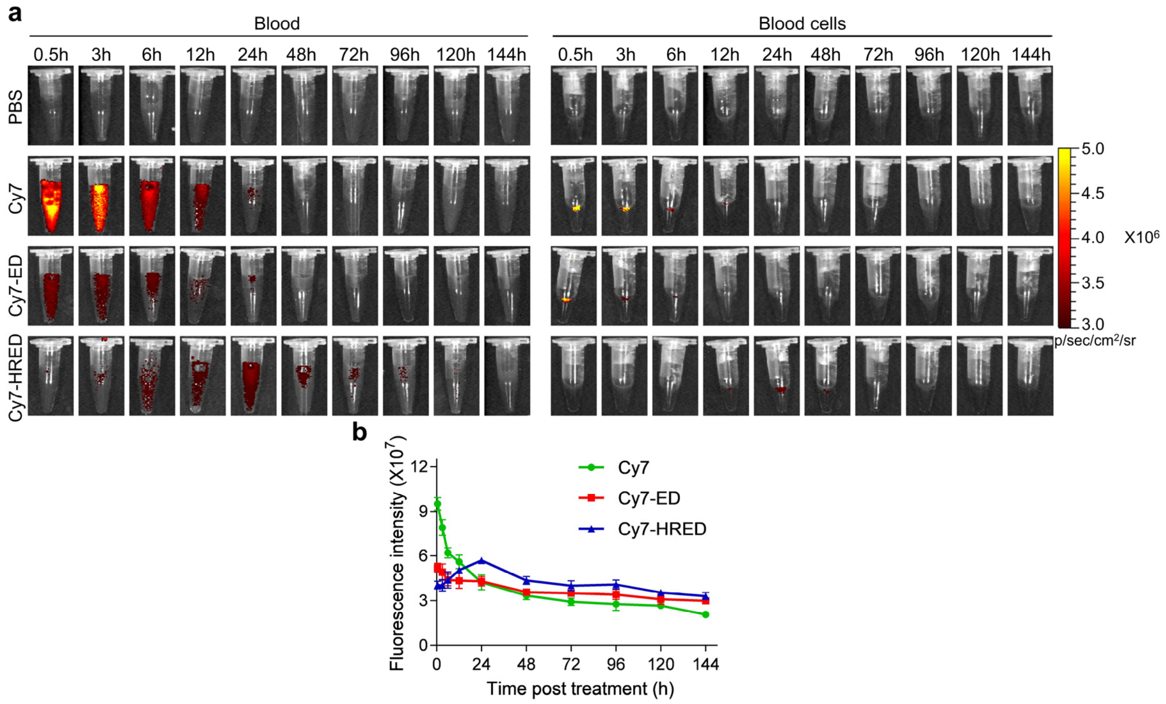

Figure S14. a) Fluorescent images of rat peripheral blood and blood cells with free Cy7, Cy7-ED and Cy7-HRED injected to rats at different time points, as determined by IVIS. b) The corresponding semiquantitative results of rat plasma in different groups. Results are represented as mean ± SD (n = 3).

2.15 Tumor targeting ability of EVs-based nanodrugs in orthotopic SKOV3-Luc ovarian cancer xenograft nude mouse model.

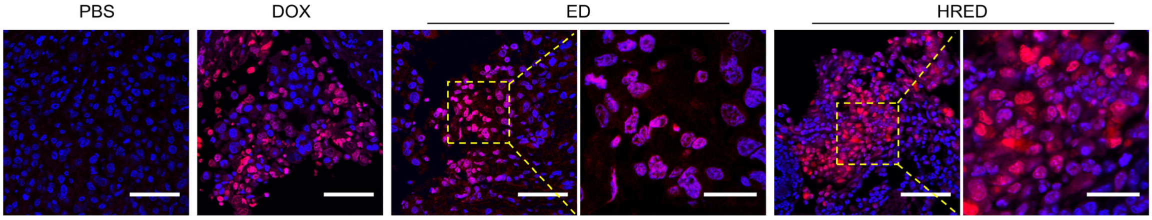

Figure S15. Confocal images of the ovarian cancer tissues from orthotopic SKOV3-Luc ovarian cancer xenograft nude mouse model after intraperitoneal injection of PBS, DOX, ED and HRED. Nucleus stained by Hoechst 33342 and red signal represented DOX. Scale bar: 50 μm in low magnification, 20 μm in high magnification.

2.16 Hemolysis test.

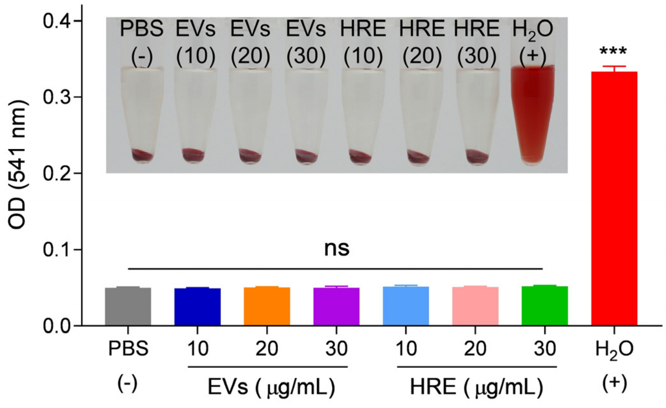

Figure S16. The images of red blood cells incubated with EVs or HRE were shown and the corresponding OD values at 541 nm of the supernatants were quantified to determinate the magnitude of red blood cell hemolysis. PBS and ultrapure water were set as a negative and positive control, respectively. Results are represented as mean ± SD (n = 3). **P* < 0.05, ***P* < 0.01, and ****P* < 0.001.

2.17 Detection of liver and kidney functions of the C57BL/6 mice receiving treatments.

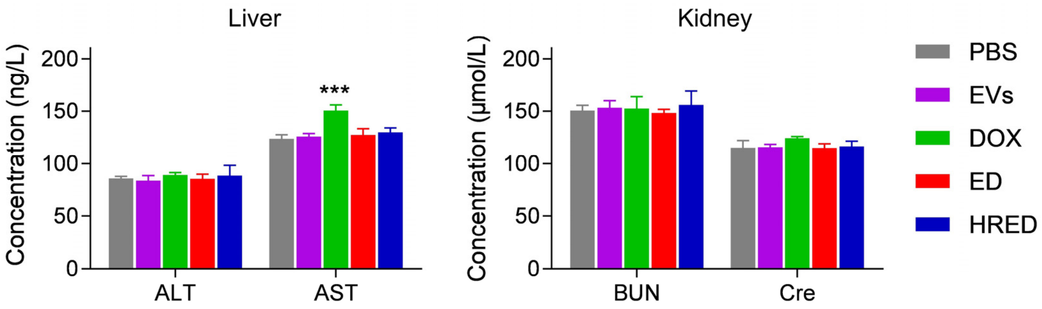

Figure S17. Serum biochemical indicators (ALT, AST, BUN and Cre) concentrations of C57BL/6 mice serum at 24 h after administration of PBS, DOX, ED and HRED (2.5 mg/kg DOX), and EVs (equivalently to HRED) via intraperitoneal injection. ALT, alanine aminotransferase; AST, aspartate aminotransferase; BUN, blood urea nitrogen; Cre, creatinine. Results are represented as mean ± SD (n = 3). *$P < 0.05$, **$P < 0.01$, and ***$P < 0.001$.

2.18 Antitumor efficacy of EVs in orthotopic SKOV3-Luc ovarian cancer xenograft nude mouse model.

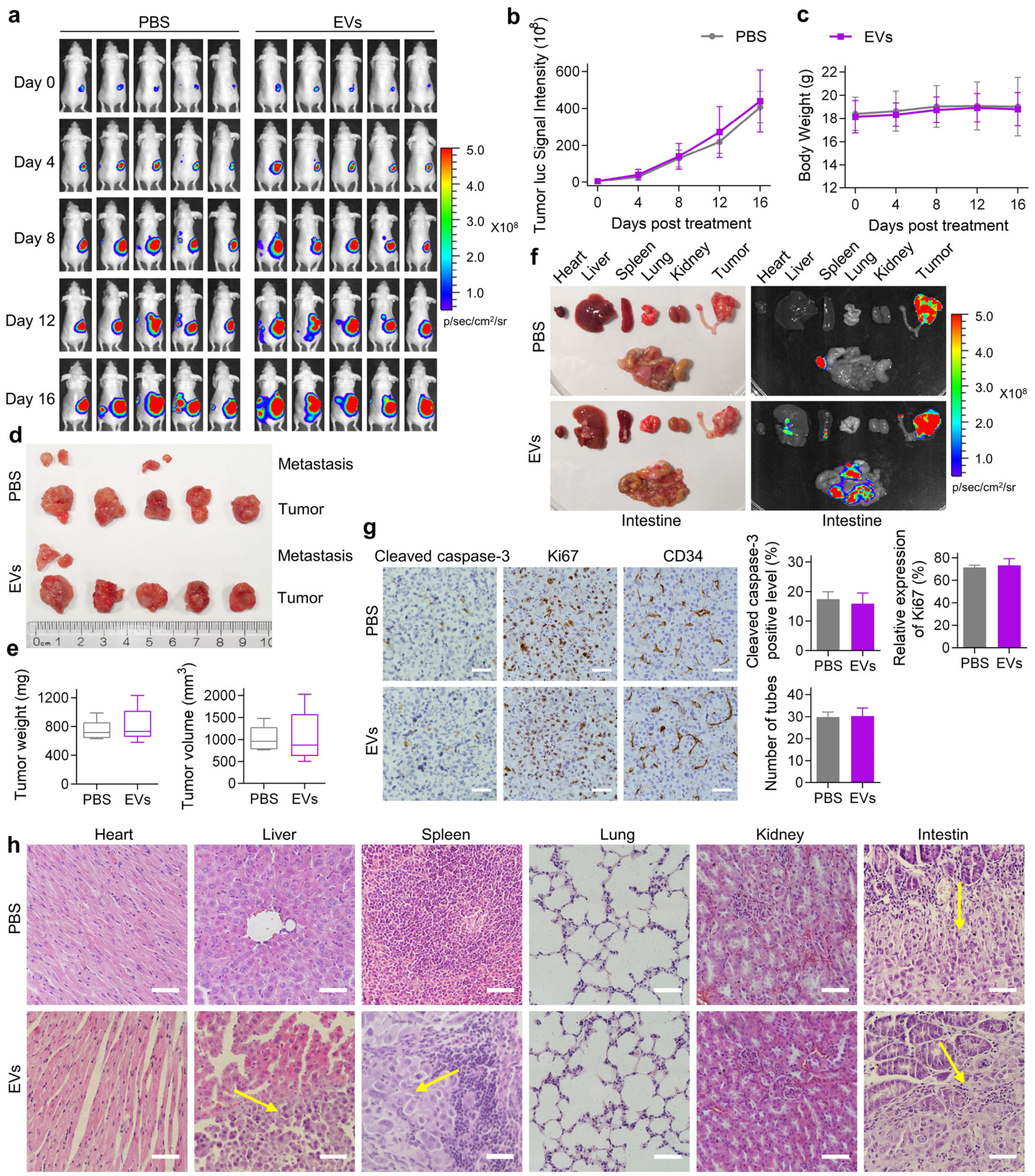

Figure S18. a) IVIS bioluminescent imaging of orthotopic SKOV3-Luc ovarian cancer xenograft nude mouse from PBS and EVs group during treatment. All mice were injected SKOV3-Luc cells into the ovaries, followed by treatment and measurement of bioluminescence after 3 days (day 0). The mice were intraperitoneally treated with PBS or

EVs (approximately 2 mg/kg) every three days. The tumor size was quantified by IVIS every 4 days. After 16 days of treatment, mice were sacrificed and the ovarian primary tumors, metastatic nodules and major organs were excised and detected for bioluminescence signals. b) The luminescent signal intensity of the mice in PBS and EVs group. c) Body weight of the mice in PBS and EVs group. d) Excised tumor of the mouse from PBS and EVs group. Scale: 1 unit=1 cm. e) Tumor weights and tumor volume of the mice in PBS and EVs group. f) Photographs and luminescent signal of major organs and tumors of mice in PBS and EVs group to detect metastatic nodules. g) Representative immunohistochemistry images and corresponding quantitative results of cleaved caspase-3 for cell apoptosis, Ki67 for cell proliferation and CD34 for the detection of endothelium vessels in tumor tissues from the orthotopic SKOV3-Luc ovarian cancer xenograft nude mouse model treated with PBS and EVs. Scale bars, 50 μm. h) Representative images of H&E organ staining from the orthotopic SKOV3-Luc ovarian cancer xenograft nude mouse model from PBS and EVs group. yellow arrows point to tumor metastasis. Scale bar: 50 μm. Results are represented as mean ± SD (n = 5). *P < 0.05, **P < 0.01, and ***P < 0.001.

2.19 Antitumor efficacy of EVs-based nanodrugs in orthotopic SKOV3-Luc ovarian cancer xenograft nude mouse model.

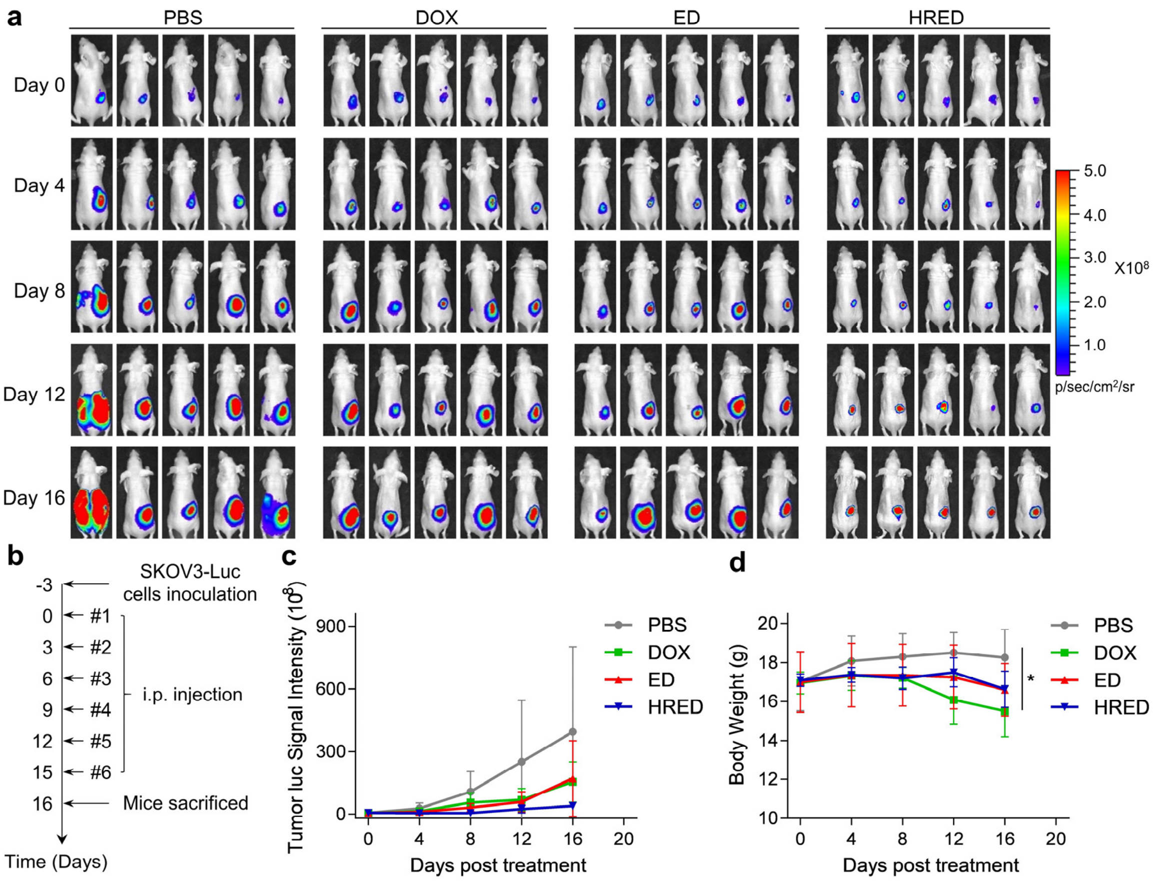

Figure S19. a) IVIS bioluminescent imaging of orthotopic SKOV3-Luc ovarian cancer xenograft nude mouse from each group during treatment. All mice were injected SKOV3-Luc cells into the ovaries, followed by treatment and measurement of bioluminescence after 3 days (day 0). The mice of the control group were intraperitoneally treated with PBS and those in the other groups with EVs (the equivalent dose with HRED), free DOX, ED or HRED (2.5 mg/kg DOX) every three days. The tumor size was quantified by IVIS every 4 days. b) Protocol for tumor implantation and treatment used in this study. c) The luminescent signal intensity of the mice in all groups. d) Body weight of the mice in all groups. Results are represented as mean ± SD (n = 5). $*P < 0.05$, $**P < 0.01$, and $***P < 0.001$.

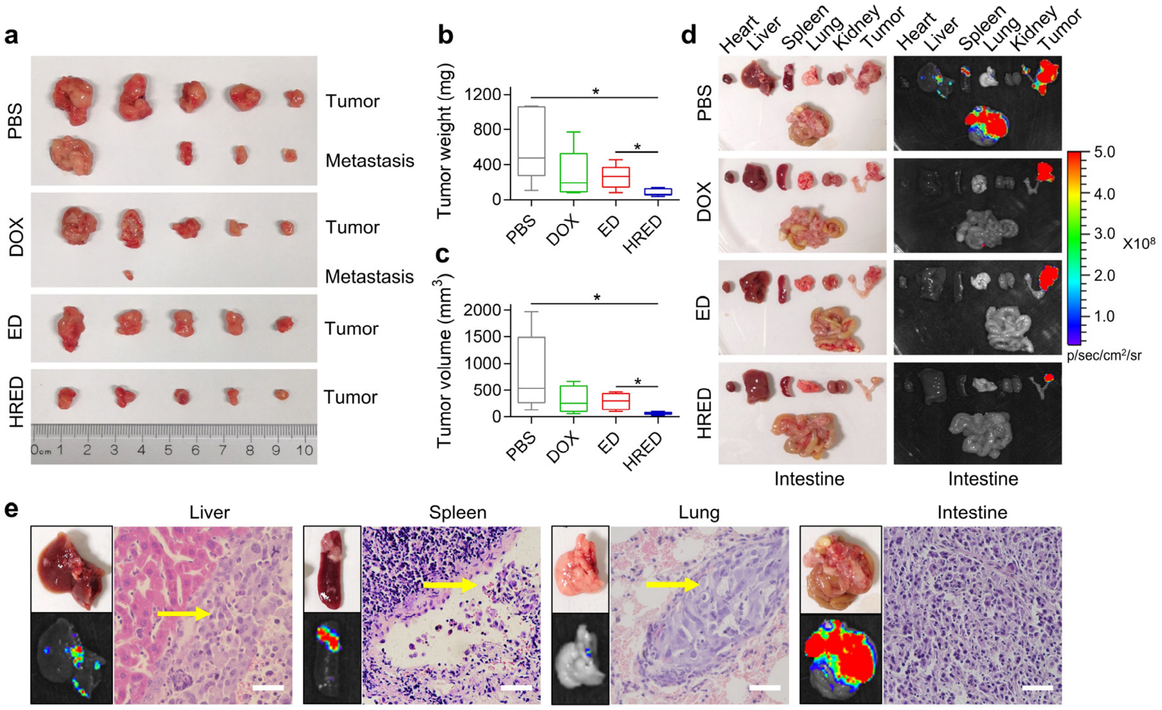

Figure S20. a) Excised tumor of the mouse from each group. Scale: 1 unit = 1 cm. b) Tumor weights of the mice in all groups. c) Tumor volume of the mice in all groups. d) Photographs and luminescent signal of major organs and tumors of mice in all groups to detect metastatic nodules. e) Images and HE staining of metastatic nodules of liver, spleen, lung and mesentery in PBS group. yellow arrows point to tumor metastasis, Scale bar: 50 μm. Results are represented as mean ± SD (n = 5). *$P < 0.05$, **$P < 0.01$, and ***$P < 0.001$.

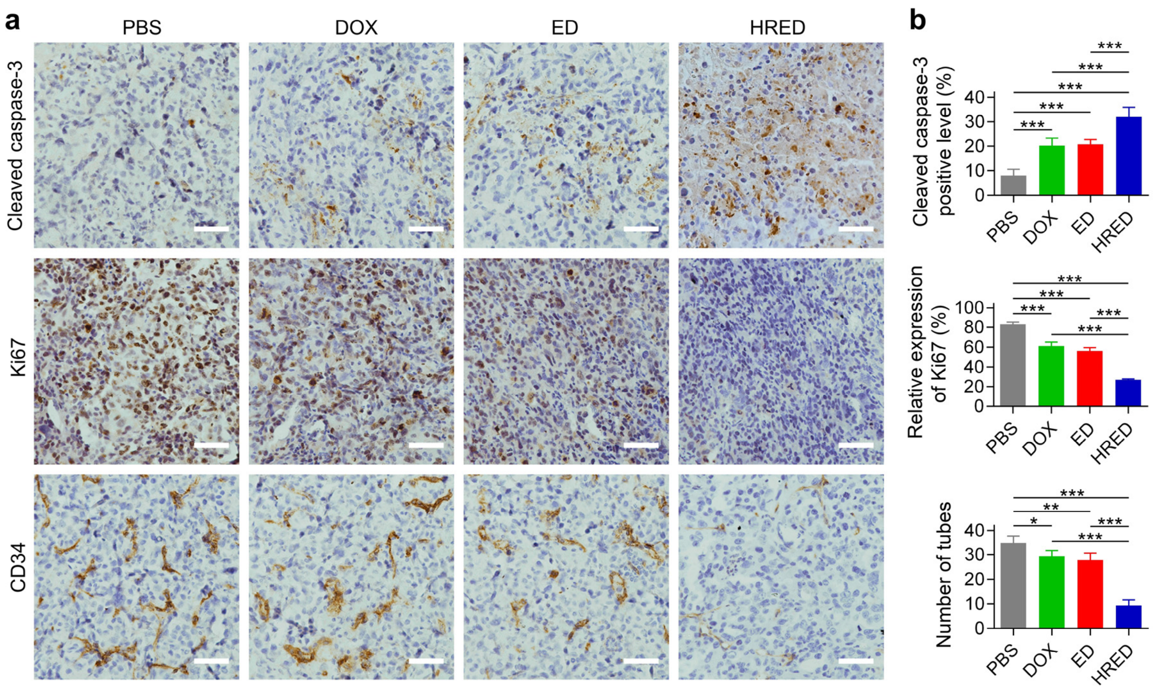

Figure S21. a) Representative immunohistochemistry images of cleaved caspase-3 for cell apoptosis, Ki67 for cell proliferation and CD34 for the detection of endothelium vessels in tumor tissues from the orthotopic SKOV3-Luc ovarian cancer xenograft nude mouse model treated with DOX, ED and HRED. Scale bars, 50 μm. b) The immunohistochemical corresponding quantitative results of cleaved caspase-3, Ki67 and CD34 in different groups. Results are represented as mean ± SD (n = 5). **P* < 0.05, ***P* < 0.01, and ****P* < 0.001.

2.20 Systemic toxicity of EVs-based nanodrugs in orthotopic SKOV3-Luc ovarian cancer xenograft nude mouse model.

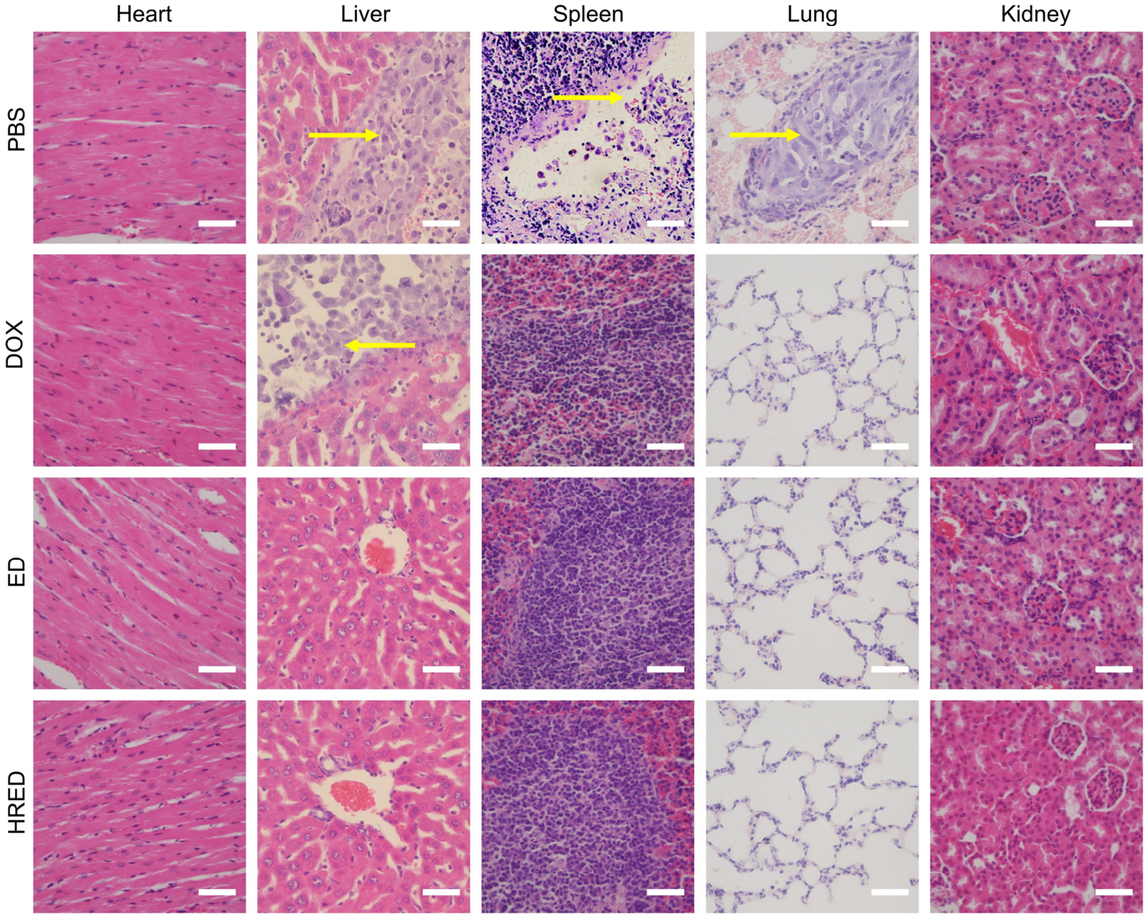

Figure S22. Representative images of H&E organ staining from the orthotopic SKOV3-Luc ovarian cancer xenograft nude mouse model from each group. yellow arrows point to tumor metastasis. Scale bar: 50 μm.

2.21 Antitumor efficacy of EVs-based nanodrugs in orthotopic SKOV3/DOX-Luc ovarian cancer xenograft nude mouse model.

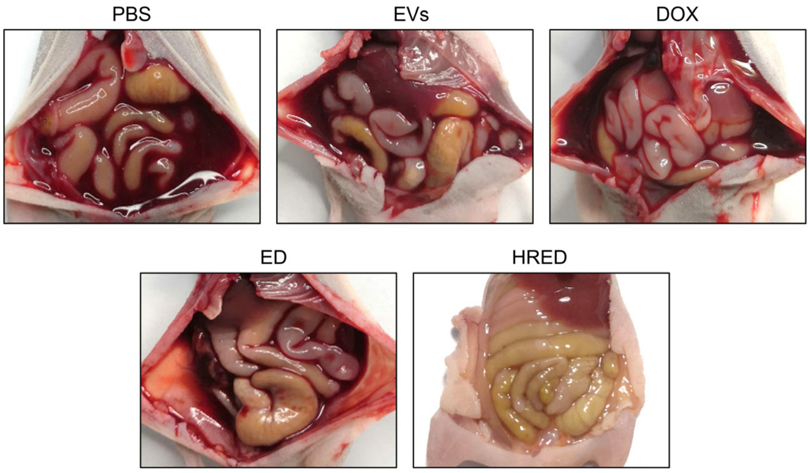

Figure S23. Representative images of malignant ascites of orthotopic SKOV3/DOX-Luc ovarian cancer xenograft nude mouse model in all groups.

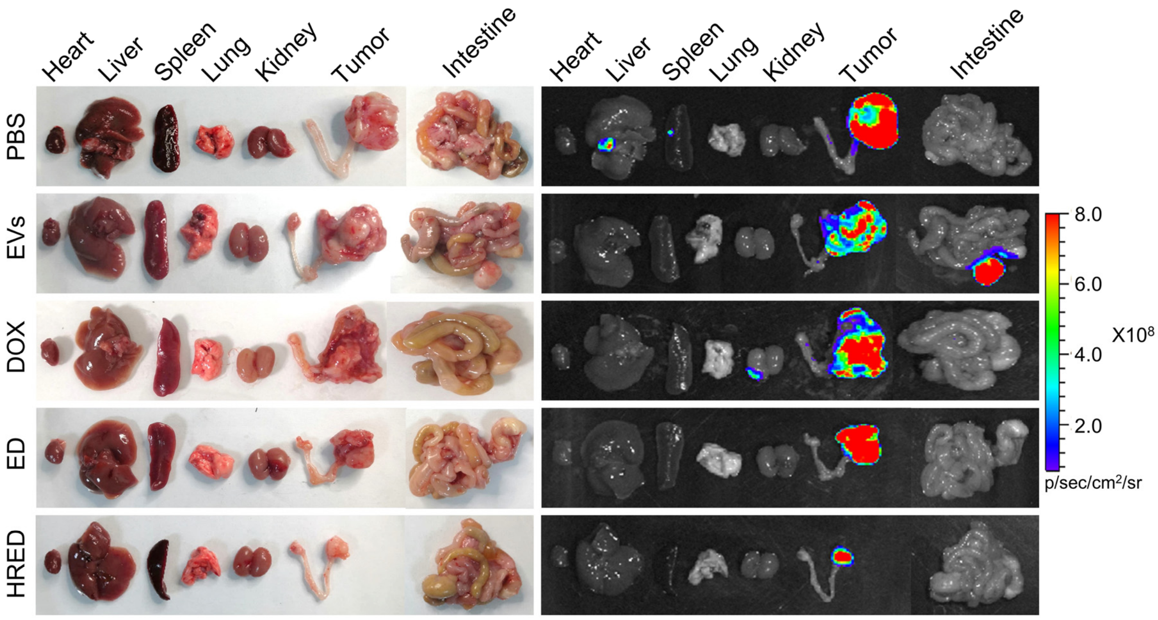

Figure S24. Representative images and luminescent signal of major organs and tumors of orthotopic SKOV3/DOX-Luc ovarian cancer xenograft nude mouse model in all groups to detect metastatic nodules.

2.22 Systemic toxicity of EVs-based nanodrugs in orthotopic SKOV3/DOX-Luc ovarian cancer xenograft nude mouse model.

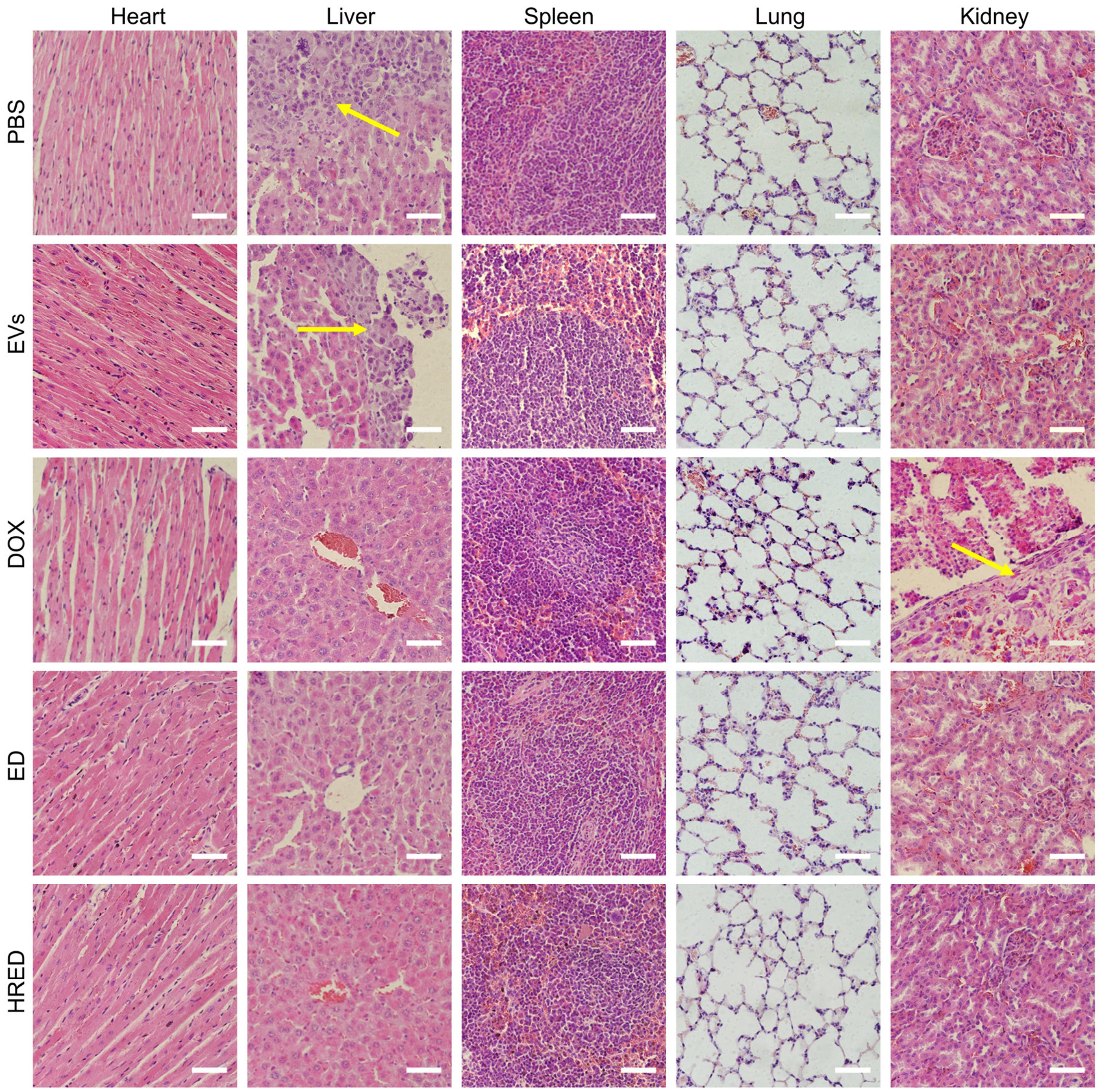

Figure S25. Representative images of H&E organ staining from the orthotopic SKOV3/DOX-Luc ovarian cancer xenograft nude mouse model from each group. yellow arrows point to tumor metastasis. Scale bar: 50 μm.

2.23 Influence of HR and DOX on protein expression in SKOV3/DOX cells.

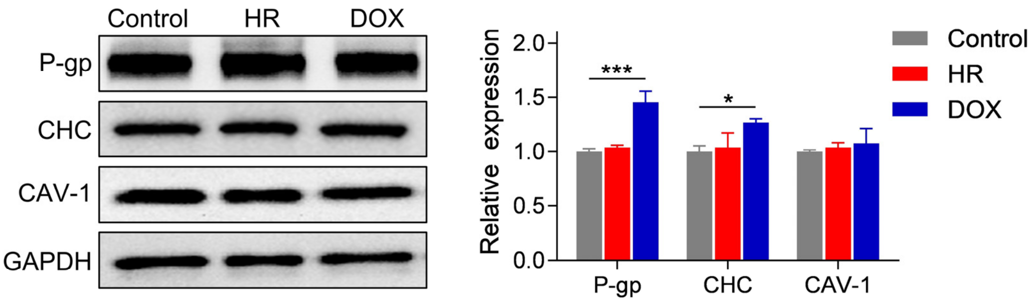

Figure S26. Protein levels and relative expression of P-gp, clathrin heavy chain (CHC) and CAV-1 in SKOV3/DOX cells after being treated with HR and DOX for 24 h. Results are represented as mean ± SD (n = 3). *$P < 0.05$, **$P < 0.01$, and ***$P < 0.001$.

2.24 Localization of P-gp and EVs-based nanodrugs in SKOV3/DOX cells.

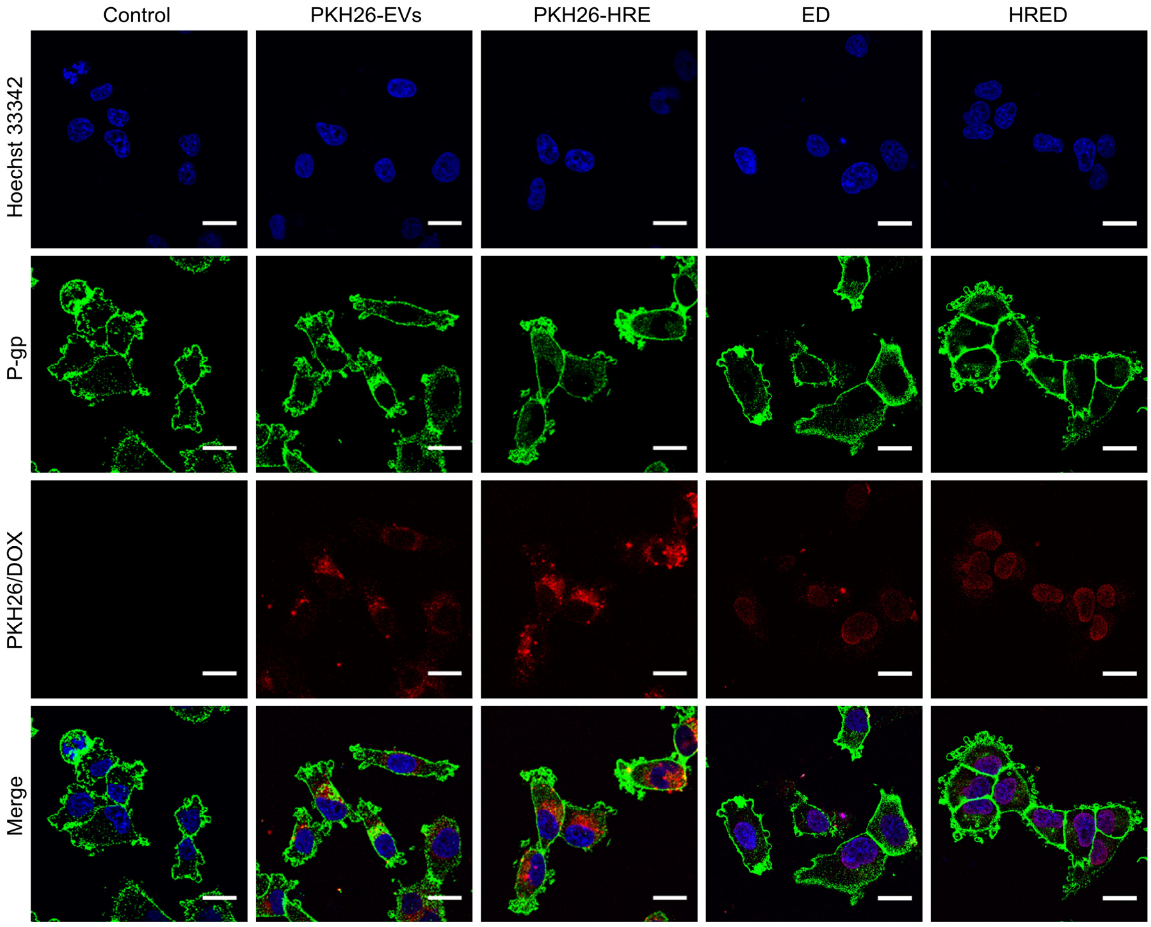

Figure S27. The intracellular localization of P-gp after treated with PKH26-EVs, PKH26-HRE, ED and HRED in SKOV3/DOX cells for 2 h, as determined by confocal microscopy. Scale bar: 20 μm.

2.25 Influence of EVs-based nanodrugs on mRNA expression in SKOV3/DOX cells.

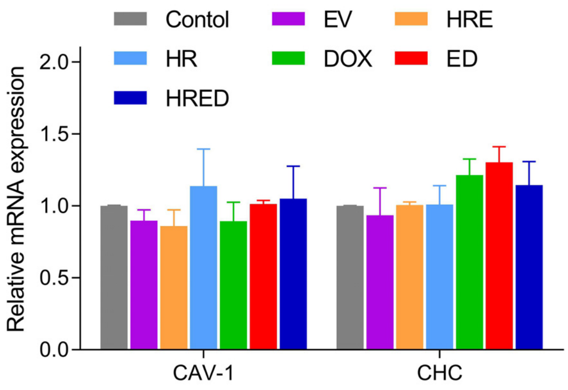

Figure S28. Relative mRNA expression of CAV-1 and Clathrin heavy chain (CHC) in SKOV3/DOX cells treated with EVs, HRE, HR, DOX, ED and HRED for 6 h. Results are represented as mean ± SD (n = 3).

2.26 Localization of CAV-1 and EVs-based nanodrugs in SKOV3/DOX cells.

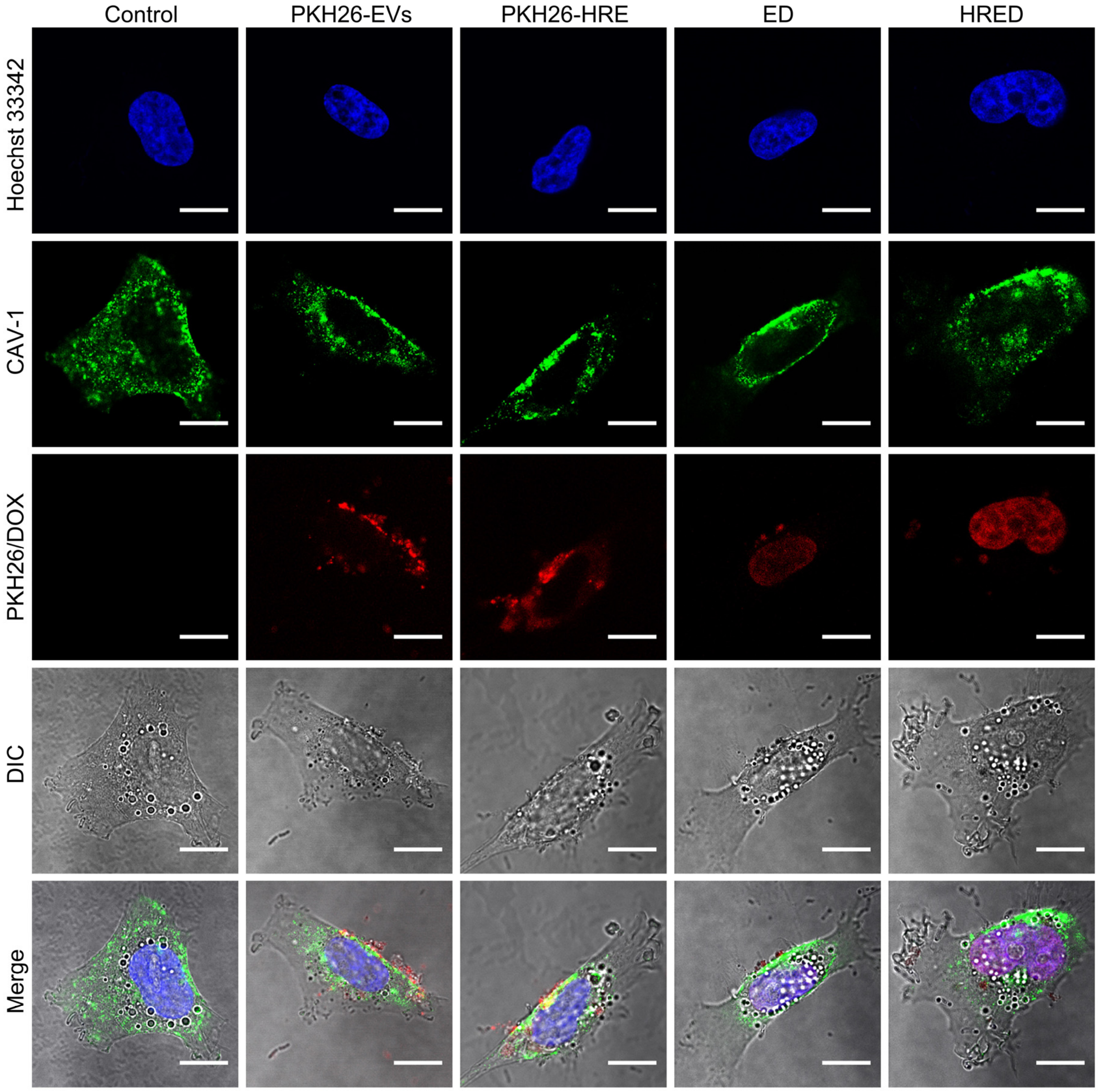

Figure S29. The intracellular localization of CAV-1 in SKOV3/DOX cells treated with PKH26-EVs, PKH26-HRE, ED and HRED for 2 h, as detected by confocal microscope. Scale bar: 10 μm.
